# Family planning and nutrition: systematic review of the effects of family planning on nutritional status of adolescent girls and women of reproductive age

**DOI:** 10.1136/bmjgh-2024-015734

**Published:** 2025-04-25

**Authors:** Ilana Rachel Cliffer, Cara Yelverton, Jingwen Dong, Matthew Dwumah-Agyen, Elisabetta Ferrero, Uttara Partap, Iqbal Shah, Wafaie Fawzi

**Affiliations:** 1 Department of Global Health and Population, Harvard University T H Chan School of Public Health, Boston, Massachusetts, USA; 2 Healthy Diets, World Vegetable Center, Chatuchak, Bangkok, Thailand; 3 Department of Epidemiology, Harvard University T H Chan School of Public Health, Boston, Massachusetts, USA

**Keywords:** Nutrition, Anaemia, Global Health, Health policy, Systematic review

## Abstract

**Introduction:**

Contraceptive use may affect women’s nutritional status through birth spacing, parity, age at first birth, menstruation changes, and increased income. To inform the integration of family planning with nutrition interventions, we synthesised evidence linking the use of family planning to nutritional outcomes in women of reproductive age (15–49 years) and adolescents (10–19 years) in low- and middle-income countries (LMICs).

**Methods:**

We searched PubMed, Embase, Web of Science and Cochrane Library for randomised controlled trials (RCTs), cluster RCTs, non-randomised trials and cohort studies published from 2000 onwards. Family planning exposure included any contraception type with no restrictions by comparison arms. Outcomes were maternal anthropometry and iron-status indicators. Random effects meta-analyses were done for comparisons with a minimum of three studies sharing intervention arms, outcomes and study design. Risk of bias and certainty of evidence were assessed.

**Results:**

Of 20 097 publications, 99 were eligible for inclusion, covering 29 outcomes and 23 interventions (eg, oral contraception, intrauterine devices (IUD)). In 28 instances, at least three studies matched on intervention arms, outcomes and study design. Meta-analysis of RCTs showed that users of hormonal IUDs had significantly higher haemoglobin than oral contraceptive users (four studies; mean difference=1.25 g/dL; 95% CI: 0.38, 2.12; certainty=very low). Users of any hormonal contraceptive had a small reduction in body mass index (BMI) compared with non-contraceptive controls (seven studies; mean difference=−0.28 kg/m2; 95% CI: −0.52 to –0.04; certainty=low); however, most samples were women with polycystic ovarian syndrome. Other relationships were very uncertain and not statistically significant.

**Conclusion:**

Evidence is weak suggesting that hormonal IUDs increase haemoglobin compared with oral contraceptives and that any hormonal contraceptive use reduces BMI. Hormonal IUDs likely limit blood loss from menstruation, allowing for higher haemoglobin than oral contraceptives. Mechanisms for lower BMI after hormonal contraceptive use remain unclear. More robust evidence is necessary to guide policy.

**PROSPERO registration number:**

This review was registered prospectively with the International Prospective Register of Systematic Review (PROSPERO ID: CD42023400069).

**WHAT IS ALREADY KNOWN ON THIS TOPIC**

- Family planning, or the use of contraceptive methods for preventing unintended pregnancies, may be related to maternal nutritional outcomes through the pathways of lengthening inter-pregnancy intervals, reducing parity, changing menstruation, delaying age at first pregnancy and increasing income and empowerment.
- Policies that integrate family planning and nutrition interventions have been proposed as a method of reducing the burden of maternal and child malnutrition in low- and middle-income countries.

**WHAT THIS STUDY ADDS**

- Limited uncertain evidence points to potential advantages to the use of hormonal intrauterine device to increase haemoglobin levels and to lower body mass index among users of any hormonal contraceptive compared with non-use of contraceptives.
- Evidence relating family planning to maternal nutrition in low- and middle-income countries is generally weak and uncertain; most studies investigated nutrition-related outcomes as side effects of contraceptive use instead of primary outcomes of interest.

**HOW THIS STUDY MIGHT AFFECT RESEARCH, PRACTICE OR POLICY**

- Additional high-quality research is needed to inform policy and practice that aim to integrate family planning and nutrition interventions.

## Introduction

Undernutrition is a public health concern among women of reproductive age (WRA) (age 15–49) and adolescent girls (10–19 years) in low- and middle-income countries (LMICs), where the combined prevalence of stunting, wasting, underweight and micronutrient deficiencies can reach 50% among WRAs^1^ and 6%–52% among adolescents.^2–4^ Additionally, over 30% of women in LMICs had anaemia in 2018.^5^ During pregnancy, mothers experience metabolic and biological adaptations to support the growth and development of the fetus,^6^ and maternal nutritional status impacts this process.^7 8^ Optimising nutritional status during this time is especially important for reducing morbidity and mortality among WRAs, adolescents and infants in LMICs.^1 9 10^

Recent literature emphasises addressing the underlying, systemic causes of suboptimal nutritional status,^11^ with knowledge and use of family planning (FP) methods as a key factor.^12^ FP methods are contraceptive methods used for preventing unintended pregnancies, attaining the specific number of offspring a woman or a couple may wish to have and spacing between pregnancies as desired. In LMICs, there is a large unmet need for FP, with 20%–58% of WRA lacking access to modern contraceptives, especially among younger women and adolescents.^13^ As such, policy advocacy groups are promoting integrated interventions of FP and nutrition to reduce maternal and infant malnutrition.^14–16^

While research has investigated relationships between FP and maternal nutritional status,^12 14^ no systematic review has synthesised the evidence and quantitatively estimated effects of FP on nutrition in LMICs. This review aims to synthesise findings on the relationship between FP and maternal nutrition, offering insights to develop a research agenda for the integration of FP with nutrition. We focus the review on maternal nutritional status given the potential it holds for influencing nutrition throughout the life cycle.

**Figure 1:**
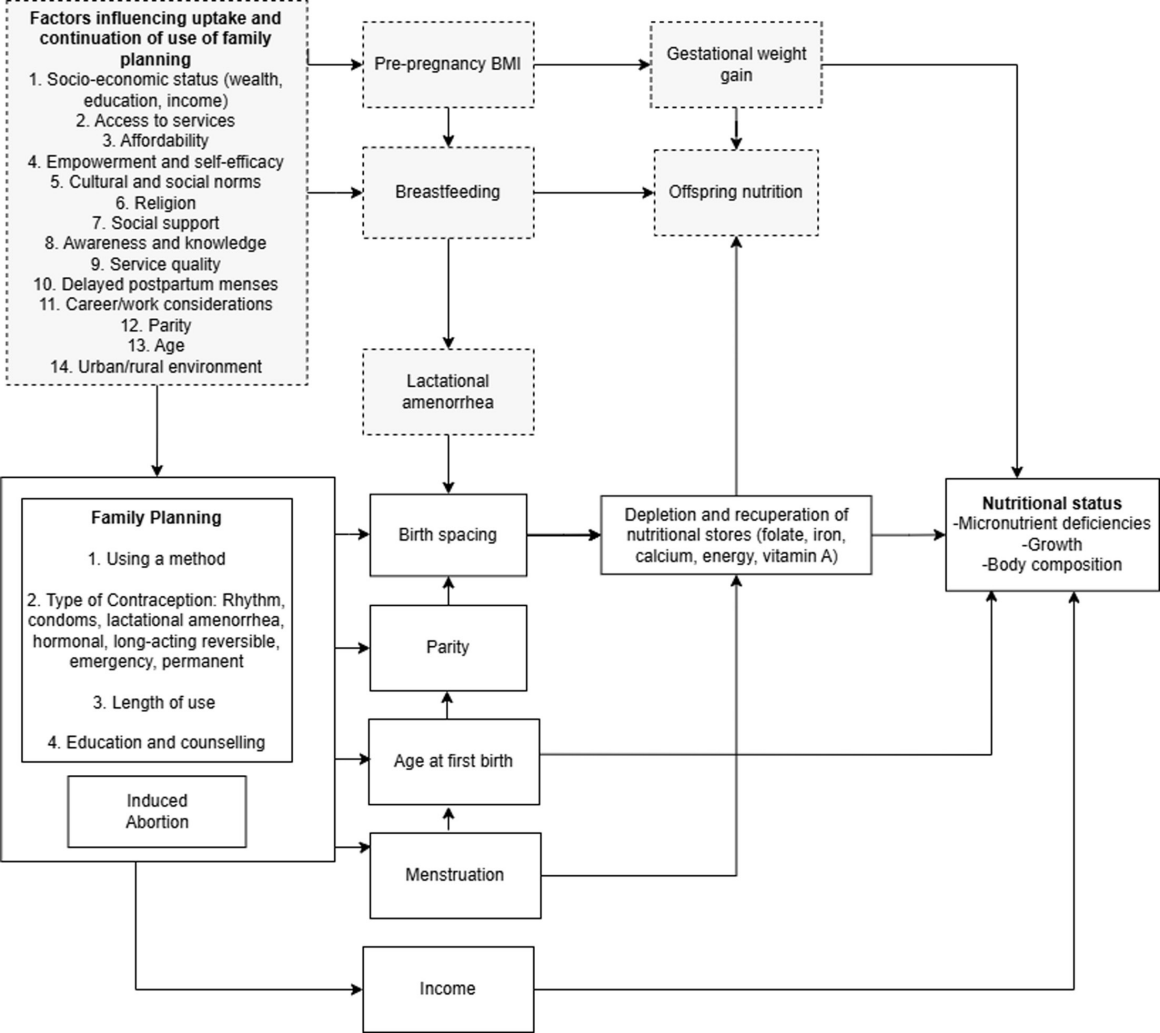
Conceptual framework depicting the potential pathways of the relationship between the uptake, type and length of use of family planning methods on maternal nutritional status. Those highlighted in grey are included for a comprehensive framework but are not included in the search strategy of this systematic review.

Five proposed pathways link FP to maternal nutritional status (figure 1): interpregnancy intervals, parity, changes to menstruation, age at first birth, and income and empowerment. Short interpregnancy intervals (18 months or less) may contribute to maternal malnutrition, allowing less time for replenishment and recuperation of nutritional stores for mothers,^17 18^ though evidence remains unclear due to lack of high-quality studies.^17 19^ FP also helps manage family size, reducing the strain of high parity on suboptimal fat accretion patterns in the body during late pregnancy and lactation^20^ and higher susceptibility to haemorrhage leading to anaemia.^21^ Contraceptive use may also influence menstrual cycles, affecting nutritional stores such as folate, iron, calcium, vitamin A and energy.^22^ Additionally, FP can delay early pregnancies, reducing health risks for adolescent mothers and their offspring.^23^ Pregnant adolescents may experience growth restriction, nutritional deficiencies and underweight, supporting the growth and development of a foetus while in a stage of rapid development themselves.^24^ Young age at first birth is also associated with lifelong socioeconomic disadvantages, which could influence maternal nutritional status.^25^ Last, the use of FP facilitates women’s work participation, income generation and intra-household allocation of resources. If resources are lacking, mothers with large families may be reluctant to prioritise their own nutritional needs.^26–28^

This systematic review examines the link between FP interventions (contraceptive uptake, method used including modern contraceptives and traditional birth spacing, and counselling) and nutritional status among WRAs and adolescent girls in LMICs.

## Methods

### Eligibility criteria

#### Study type

Eligible studies included randomised controlled trials (RCTs), cluster RCTs, non-randomised trials and cohort studies. Quasi-experimental designs (non-randomised trials and cohort studies) were included if concurrent comparator groups were examined. Studies could be research investigations or evaluations of population-based programmes.

#### Participants

We included studies that measured nutritional status outcomes among WRA (15–49 years) and adolescent girls (10-19) (pregnant or non-pregnant) from LMICs, defined by the World Bank country classification for the 2023 fiscal year.

#### Types of interventions or exposures

The main intervention (RCTs or quasi-experimental studies) or exposure (cohort studies) of interest was FP; we also included studies examining induced abortion methods as a key form of fertility regulation. We defined exposure to FP and related practices by three categories: type of contraception, use of contraception (uptake) and duration of use of contraception. Type of contraception was defined as^29^ : oral contraceptives (combined oral contraceptive pills and progestogen-only pills), implants, injectables, contraceptive patches, vaginal rings, intrauterine devices (hormonal and non-hormonal), emergency contraception, condoms (male and female), lactational amenorrhea method (LAM), sterilisation (vasectomy and tubal ligation), standard days method, rhythm/calendar method, basal body temperature method, withdrawal and sympto-thermal method. We also considered FP education, counselling, commodity provision and other interventions aimed at increasing uptake of FP methods. We use the term FP for contraceptive use irrespective of marital status or sex of the user and the primary reason for use.

#### Comparator

We did not restrict our search by comparator. Comparator groups could include: no FP intervention delivered (control group), standard of care/routine care, a different FP intervention (eg, hormonal vs non-hormonal or oral contraceptive vs hormonal intrauterine device (IUD), etc), education only or placebo.

#### Outcomes

Outcomes were maternal nutritional status indicators, including: haemoglobin, ferritin or haematocrit levels; body composition metrics (ex: body mass index and lean and fat mass); anthropometry (ex: weight, height, waist-hip ratio, abdominal circumference, skinfolds of biceps, triceps, subscapular, suprailiac and thigh); and adolescent growth indicators (z-scores, growth trajectories).

Micronutrient status indicators (ie, deficiency, insufficiency or sufficiency in vitamin and mineral levels as measured in blood plasma and urine assays of women) are also outcomes of nutritional status, but we limit the scope of blood level measures to iron-level indicators for this review given the priority of addressing factors that contribute to anaemia.

### Search methods

#### Electronic searches

Literature search was conducted using PubMed, Embase, Web of Science core collection and Cochrane Library (Cochrane Central Register of Controlled Trials) on 28 February 2023. We focused the review on peer-reviewed literature to keep the highest standard of rigour in evidence synthesis. Key terms included those relevant to FP, induced abortion, nutritional status, the population of interest and the setting. The full search strategy is presented in online supplemental table 1. Search terms included micro- and macro-nutrients; however, studies related to these outcomes were later excluded at the full text stage, as we prioritised outcomes related to haemoglobin, body composition and anthropometry. Future reviews may focus on micro- and macro-nutrient status as outcomes.

10.1136/bmjgh-2024-015734.supp1 Supplementary data

#### Search restrictions

English language studies and studies published from the year 2000 onwards were included.

### Study selection

All literature searched through bibliographic databases was uploaded to Covidence review management software (Veritas Health Innovation, Melbourne, Australia). Covidence was used to manage study selection, and Microsoft Excel was used for data extractions. Based on eligibility criteria, two authors from the list of co-authors (IRC, CAY, JD, MDA, UP) independently assessed potential studies for inclusion, in the following sequence: (1) search results were merged; (2) duplicate records were removed; (3) titles and abstracts were screened, and studies clearly not meeting selection criteria were removed, while those where potential eligibility was unclear were included for full text review; (4) disagreements in title and abstract review were resolved via discussion with at least three co-authors; (5) full texts were retrieved, reviewed and included if they met selection criteria; (6) disagreements in full text review were resolved through discussion or consultation with a third reviewer; and (7) inclusion of studies was finalised and a Preferred Reporting Items for Systematic Reviews and Meta-Analyses (PRISMA diagram prepared.

### Data extraction and management

Two reviewers from the list of co-authors (IRC, CAY, JD, MDA, EF) extracted data from the eligible literature independently using a prespecified tailored data extraction form developed by the authors. Prior to conducting extractions, a pilot test of two included studies was conducted to ensure consistency among the reviewers. Data extracted included author name, publication year, study population and setting, study design, participant characteristics, interventions or exposures and comparators, outcomes and analytical strategy. A comprehensive list of data extracted is provided in online supplemental table 2. Both crude and adjusted effect estimates were extracted, when available. Discrepancies between the two reviewers extracting each study were resolved through discussion and consensus and usually consisted of small errors in transferring numbers. In the case of unsolved disagreements, a third reviewer made the final decision.

### Risk of bias assessment

For RCTs, two authors from the list of co-authors (IRC, CAY, JD, MDA, EF) independently assessed risk of bias for each study using the Risk of Bias-2 (ROB-2) tool for RCTs or ROB-2 Cluster Randomised Trials (ROB-2-CRT) for cluster randomised studies.^30^ For non-randomised trials, the authors used the Risk Of Bias in Non-randomised Studies – of Interventions (ROBINS-I tool)^31^ to assess risk of bias, and for observational studies, the authors used the Risk Of Bias in Non-randomised Studies – of Exposures (ROBINS-E) tool.^32^

### Data synthesis

We conducted narrative, quantitative syntheses of study results and considered meta-analyses if there were at least three studies of the same study design that shared similar outcomes and intervention arms. We estimated effects using random effects meta-analyses in Stata 17 (StataCorp, LLC, College Station, Texas, USA).^31^ Separate meta-analyses were conducted for combinations of outcome, intervention and study design matches. We prioritised comparisons for which general categories of contraception were available to compare (eg, hormonal contraceptives, non-hormonal contraceptives, no contraception) and those among RCTs; however, we conducted some comparisons for non-randomised trials where a sufficient number of RCTs were unavailable. Depending on availability, we report both intention-to-treat and per-protocol results. Additional levels of stratification (by study population, region, etc) were not possible due to the small number of studies in each stratum. In addition, we had intended to examine the duration of use and adherence to intervention as a factor in exposure to FP; however, no available studies included this as part of their study design. For performed meta-analyses, no adjusted results were available; we thus present only unadjusted results in our meta-analyses. All studies included in meta-analyses were individually randomised, so cluster-adjusted effect estimates are not reported. If studies reported a mean and SE, SE was converted to SD to align with other studies for meta-analysis using the equation: $\text{SD = SE × }\sqrt{\text{N}}$

$$SD=SE\times \sqrt{N}$$

Where possible, we conducted sensitivity analyses including only studies with low risk of bias. We report relative risks or ORs for categorical data and mean differences or standardised mean differences where possible for continuous data, along with respective 95% CIs. Funnel plots and Egger’s test were used to assess the presence of publication bias; however, funnel plots were not presented unless a minimum of 10 studies were included in the comparison. Assessment of statistical heterogeneity among studies was done using a combination of visual inspection of forest plots including the overlap of CIs among studies and the I^2^ value (calculated as I^2^=100% x (Q-df)/Q; where Q is Cochrane’s heterogeneity statistic and df is the df). I^2^ values were interpreted as: <25% no heterogeneity, 25%–49% low heterogeneity, 50%–74% moderate heterogeneity and ≥75% high heterogeneity.^33^

### Evaluation of certainty of evidence

Quality of the pooled estimates for each outcome was assessed through the GRADE approach, using GRADEPro GDT.^34^

### Registration and reporting

This review was registered prospectively with the International Prospective Register of Systematic Review (PROSPERO ID: CD42023400069) and was conducted and reported according to the PRISMA 2020 guidelines.^35^

### Patient and public involvement

The content of this review was determined by the identified need for evidence connecting FP interventions to maternal nutritional status. It was indirectly informed by experiences of the public regarding the available evidence for the integration of FP and nutrition programmes—an endeavour being proposed by governments in several LMICs.

## Results

### Included studies

**Figure 2:**
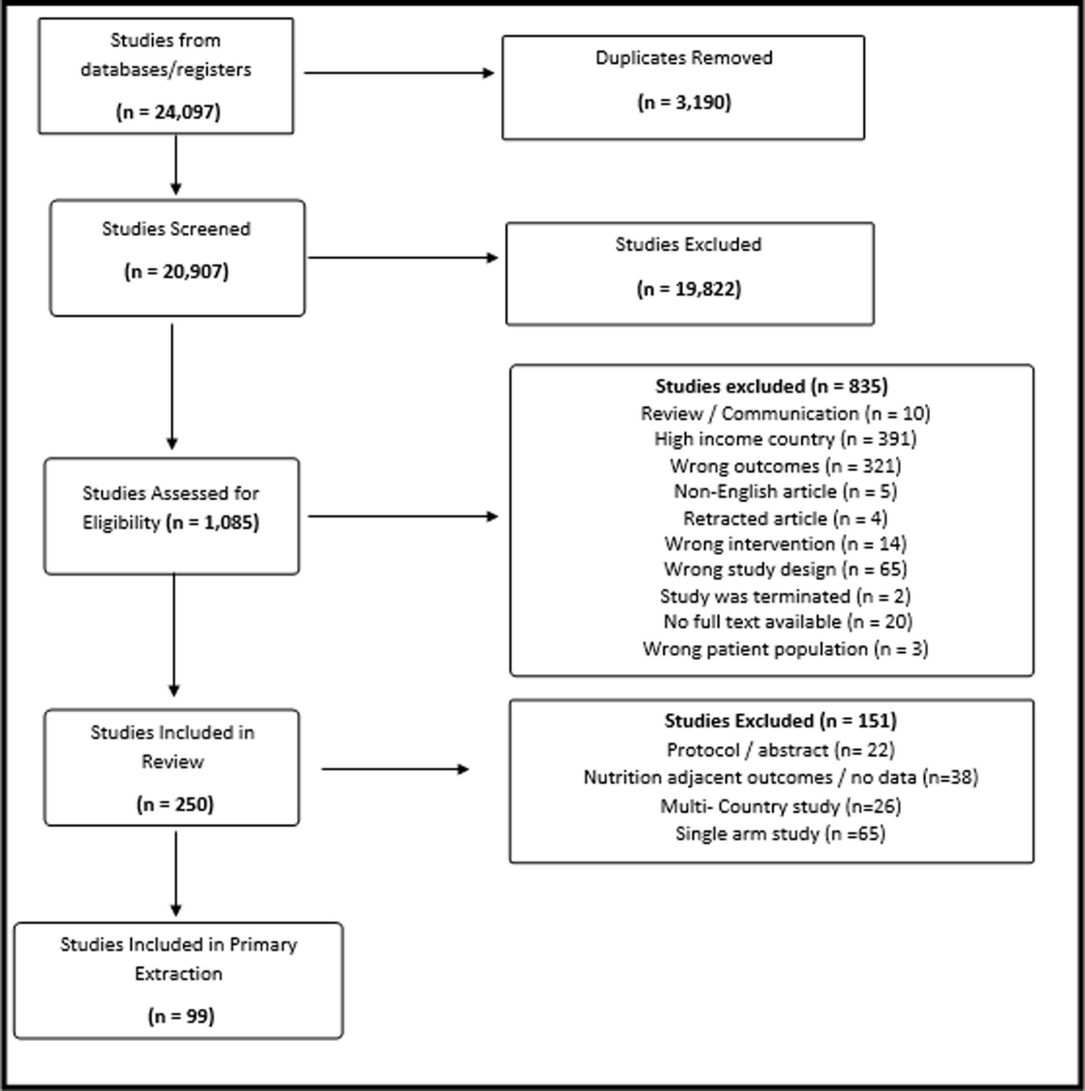
Preferred Reporting Items for Systematic Reviews and Meta-Analyses (PRISMA) flow diagram of included and excluded studies of this systematic review of contraceptive use and induced abortion and their impact on nutritional status.

The PRISMA flow diagram (figure 2) shows the information flow through each stage of study selection. After initial database searches, titles and abstracts of 20 907 studies were reviewed once duplicates were removed. Of those, 1085 studies were put forward for full text review and a final 250 were deemed eligible for extraction based on initial selection criteria. Secondary selection criteria limited extractions to studies with more than one arm, haemoglobin and anthropometry outcomes only, and studies for which data were clearly presented from LMICs, further restricting the sample of eligible studies to 99.

### Study characteristics

Online supplemental table 3 shows the basic characteristics of extracted studies. Of the 99 extracted studies, 63 were RCTs, 18 were non-randomised trials, 14 were prospective cohort studies and the last four were retrospective cohort studies. A total of 89 283 participants were enrolled in the 99 studies combined. Several of the study populations were women with polycystic ovarian syndrome (PCOS), hypertension, HIV or other syndromes.

Online supplemental table 4 shows a breakdown of the interventions or exposures covered by the extracted studies. Interventions and exposures varied widely, and most studies compared different contraceptive interventions to each other. The most common were oral contraceptives (55 studies), hormonal IUD (26 studies) and non-hormonal IUD (19 studies). Twenty of the studies had a comparator group with no contraceptive intervention; 11 of these had no alternative intervention at all, and the remaining nine had either supplementation with micronutrients, thermal balloon ablation or a non-contraceptive drug as the comparison.

Outcomes found in the extracted studies are displayed in online supplemental table 5. A total of 219 outcomes were reported for the 99 included studies (studies reported multiple outcomes). Broad categories of outcomes included body weight and composition measures (BMI, weight, fat and lean mass), anthropometry (circumferences, waist-hip ratios), and measures related to anaemia (haemoglobin, haematocrit, ferritin). Similar outcomes were often measured in different ways, restricting the number of direct comparisons possible among studies. Most outcomes were assessed between 1 and 12 months postintervention or exposure to contraceptive methods, although five studies had a 2-year follow-up, and two had a 5-year follow-up. The studies with longer duration of follow-up did not meet the requirements to be included in meta-analyses. In addition, data on exposure to contraceptives prior to inclusion in the studies were limited to a maximum of 3 months pre-inclusion across all studies.

Included studies were based in a range of countries (online supplemental table 6), with every subregion (East Asia and Pacific, Europe and Central Asia, Latin America and the Caribbean, Middle East and North Africa, North America, South Asia and Sub-Saharan Africa) represented by at least one country. Many studies were carried out in Brazil, Turkey and Iran. Online supplemental figure 1 is a map of study locations.

Risk of bias was assessed separately by outcome for each study, so studies with multiple outcomes have multiple assessments (online supplemental table 7). Studies were judged to be at low risk of bias for only 26 out of 219 total outcomes assessed (12%). Most were moderate risk/some concerns (n=77), followed by very high/critical risk (n=58). Finally, 56 outcomes were of very high/serious risk of bias, and two had no information by which to judge. Generally, all outcomes for a study were of the same risk of bias based on the study design and data collection procedures; however, a few studies had outcomes of differing risk of bias due to differences in the reporting of separate outcomes (eg, some self-reported, others measured using direct clinical observations).

### Selection of comparisons

Table 1 lists all combinations of outcomes, study designs and interventions with a minimum of three studies available. While 28 comparisons were possible, we prioritised outcomes with the least potential for confounding, information (measurement) bias and selection bias, for meta-analyses. We did not conduct meta-analyses with abortion interventions or exposures, as the eight abortion studies included were comparisons of different abortion methods to each other and were thus not generally informative in answering our research question. We focused on BMI, haemoglobin and weight changes, rather than absolute weight, which is not corrected for height or baseline measurements. We chose to examine the more general comparisons of groups of contraceptive types, rather than the comparisons of subtypes (eg, we prioritised all hormonal contraceptives as a group over oral contraceptives or hormonal IUDs separately). Where possible, we chose to undertake the meta-analyses among RCTs, but in the case of haemoglobin, broader comparison of contraceptive types was only possible with non-randomised trials. While we aimed to use adjusted results for our meta-analyses, none of the papers for which meta-analyses were possible reported adjusted results for our outcomes of interest. Ultimately, we conducted meta-analyses using the unadjusted results presented for the effects of hormonal IUD versus oral contraceptives on haemoglobin status; hormonal vs non-hormonal contraceptive on haemoglobin status; hormonal contraceptive versus non-contraceptive users on BMI; and any hormonal versus non-hormonal contraceptive use on BMI, weight gain and weight loss.

**Table 1:** List of comparisons for meta-analyses, matching study designs, interventions and outcomes

Outcome	Study design	Comparison	Number of studies
Abdominal circumference	Non-randomised trial	Hormonal contraceptive versus non-hormonal contraceptive	3
BMI	RCT	**Hormonal contraceptive versus any non-contraceptive**	**7**
		**Hormonal contraceptive versus non-hormonal contraceptive**	**3**
		Oral contraceptive versus other oral contraceptive	9
		Oral contraceptive versus oral contraceptive+other drug	4
		Oral contraceptive versus supplement (no contraception)	3
		Oral contraceptive versus non-contraceptive drug	3
	Non-randomised trial	Hormonal contraceptive versus any non-contraceptive	3
		**Hormonal contraceptive versus non-hormonal contraceptive**	**6**
		Oral contraceptive versus other oral contraception	6
	Cohort study	Hormonal contraceptive versus non-hormonal contraceptive	3
Fat and lean mass	Non-randomised trial	Hormonal contraceptive versus non-hormonal contraceptive	3
Haemoglobin	RCT	Abortion drug versus other abortion drug	5
		**Hormonal IUD versus oral contraceptive**	**4**
	Non-randomised trial	Hormonal contraceptive versus non-hormonal contraceptive	**3**
Waist-hip ratio	RCT	Hormonal contraceptive versus any non-contraceptive	4
		Oral contraceptive versus other oral contraceptive	5
Weight	RCT	Hormonal contraceptive versus non-hormonal contraceptive	3
		Oral contraceptive versus oral contraceptive	6
	Non-randomised trial	Hormonal contraceptive versus non-hormonal contraceptive	6
		Injectable versus non-hormonal IUD	
	Cohort study	Hormonal versus non-hormonal contraceptive	3
Weight change	RCT	Oral contraceptive versus oral contraceptive	3
Weight gain	RCT	**Hormonal contraceptive versus non-hormonal contraceptive**	**3**
		Hormonal IUD versus oral contraceptive	3
		Oral contraceptive versus oral contraceptive	5
Weight loss	RCT	**Hormonal contraceptive versus non-hormonal contraceptive**	**3**

Note: Comparisons highlighted in bold were conducted, and results presented within this manuscript. Comparisons not in bold were not conducted as meta-analyses due to either lack of heterogeneity of the two interventions being compared (eg, different types of oral contraceptives compared with each other) or outcomes that were not prioritised for this particular review.

BMI, body mass index; IUD, intrauterine device; RCT, randomised controlled trial.

### Effects of contraceptive use on haemoglobin

#### Hormonal intrauterine device (IUD) versus oral contraceptives

**Figure 3:**
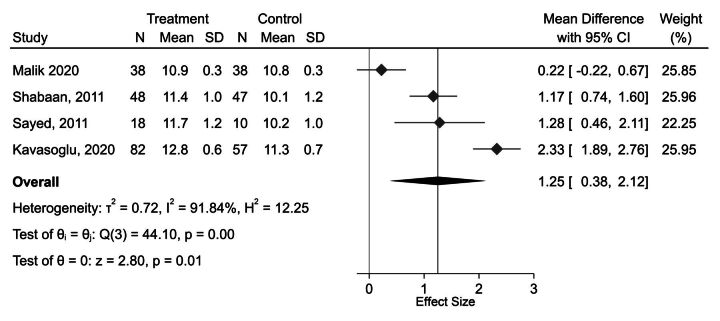
Forest plot presenting the random-effects meta-analysis of randomised controlled trials reporting the impact of hormonal intrauterine devices (treatment) compared with hormonal oral contraceptives (control) on haemoglobin (g/dL).

The evidence is very uncertain about the effect of using a hormonal IUD compared with oral contraceptives on haemoglobin. Four RCTs compared hormonal IUDs to oral contraceptives: Kavasoglu, Malik, Sayed and Shabaan^36–39^ Kavasoglu and Malik intervened with norethisterone acetate (NETA; a progesterone-only contraceptive pill),^36 37^ while Sayed 2011 and Shabaan 2011 used a low-dose combined oral contraceptive tablet.^38 39^ The random-effects meta-analysis results are presented in figure 3; a positive mean difference in haemoglobin was found among those using hormonal IUD compared with those using oral contraceptives (mean difference=1.25 g/dL; 95% CI: 0.38, 2.12; p=0.010; I^2^=92%; Egger’s test p-value=0.996; GRADE=very low). Baseline haemoglobin was balanced across the study arms in all studies except Kavasoglu,^36^ in which the baseline haemoglobin level was slightly higher in the levonorgestrel intrauterine device (LNG-IUD arm) (11.03 g/dL) compared with the oral contraceptive arm (10.56 g/dL); however, the difference from baseline to endline was much greater in the LNG-IUD arm than the oral contraceptive arm, so this difference is unlikely to have skewed the results of the meta-analysis. All studies used intention-to-treat analysis. Sayed was judged as high risk of bias,^38^ and all others had some concerns.

#### Hormonal contraceptives versus. non-hormonal contraceptives

Several studies compared hormonal contraceptives to non-hormonal contraceptives with the outcome of haemoglobin but were not included in the meta-analyses due to lack of sufficient comparisons and, in the case of the non-randomised trials, insufficient data. Four studies (two RCTs, one non-randomised trial and one cohort study) found that haemoglobin was higher in the hormonal contraceptive group (LNG-IUD^40–42^ or vaginal ring)^43^ compared with the non-hormonal contraceptive group (copper IUD). Two additional non-randomised trials and one cohort study found no difference in haemoglobin between those using hormonal contraception (EE/DRSP)^44^ or LNG-IUD^45 46^ versus non-hormonal IUDs.

Two studies investigated the effects of hormonal vs non-hormonal contraceptive methods on ferritin and haematocrit as outcomes. One study^47^ found no difference in ferritin with use of hormonal IUD compared with non-hormonal IUD, and the second^42^ found higher ferritin and haematocrit when hormonal IUD was used compared with non-hormonal IUD. Finally, ferritin was higher in the hormonal IUD group in two studies for which the comparison was oral contraception,^38 39^ but comparing the vaginal ring to oral contraception showed no difference.^48^

### Effects of contraceptive use on body mass index (BMI)

#### Hormonal contraceptive versus non-contraceptive

**Figure 4:**
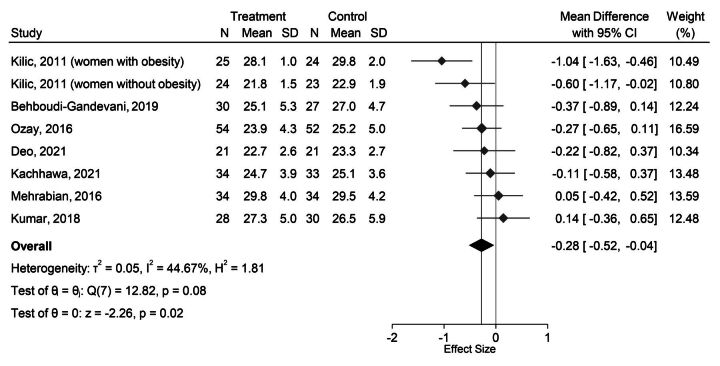
Forest plot presenting the random-effects meta-analysis of studies reporting the impact of any hormonal contraceptive method (treatment) compared with controls using no contraceptives on body mass index (BMI) (kg/m^2^).

The available evidence suggests that using hormonal contraceptives compared with not using contraceptives results in a slight reduction in BMI. Meta-analyses of the seven studies indicated that those using any hormonal contraceptive had lower BMI (mean difference=−0.28 kg/m^2^; 95% CI: −0.52 to –0.04; p=0.020; I^2^=45%; Egger’s test p value=0.207; GRADE=low) than those using non-contraceptive interventions (figure 4). Each of the seven studies used oral contraceptives as the intervention arm. Non-contraceptive interventions included phlebotomy,^49^ non-contraceptive drugs (metformin)^50–53^ and micronutrient supplementation.^54 55^ Five of the seven studies focused on populations with PCOS.^49 51–53 55^ Examination of baseline BMI in each study arm showed that all relevant study arms were balanced. Six of the studies reported intention-to-treat analyses, and one^54^ reported per protocol. Three of the studies had low risk of bias overall,^49 53 54^ and the remaining four had moderate risk of bias with some concerns.^50–52 55^ We conducted sensitivity analyses of the three studies with low risk of bias. The results showed a mean difference in BMI of −0.13 kg/m^2^ (95% CI: −0.41, 0.15). There was minimal variation between these studies, as indicated by a very low heterogeneity value (I^2^=0.0%).

#### Hormonal contraceptive versus non-hormonal contraceptive randomised controlled trials (RCTs)

The evidence from RCTs is very uncertain about the effect of using hormonal contraceptives compared with non-hormonal contraceptives on BMI. Three RCTs examined the effects of hormonal contraceptives on BMI compared with non-hormonal contraceptives. Beksinska compared the depot-medroxyprogesterone acetate (DMPA) injection and the LNG-IUD to a copper IUD,^56^ Bilgehan compared the LNG-IUD to a copper IUD,^57^ and Zueff compared the LNG-IUD to a non-hormonal contraceptive of the participants’ choice (either male condom or the copper IUD).^58^ Bilgehan and Beksinska reported intention-to-treat analyses,^56 57^ and Zueff reported per-protocol.^58^ All studies presented unadjusted results and were balanced at baseline between groups for BMI.

As Beksinska compared two different hormonal contraceptives against the non-hormonal IUD,^56^ we conducted two separate meta-analyses. The first random-effects meta-analysis used the LNG-IUD arm and suggested no meaningful difference in BMI among those who used hormonal contraceptives versus those who used non-hormonal contraceptives, and there was significant heterogeneity among the studies (mean difference=−0.17 kg/m^2^; 95% CI: −0.52, 0.20; p=0.370; I^2^=74%; Egger’s test p value=0.0791; GRADE=very low) (online supplemental figure 2a). Egger’s test suggested there was no publication bias, although this test should be interpreted with caution given that there are only three studies in the meta-analysis. Risk of bias assessments found that Beksinska had a high risk of bias,^56^ while both Bilgehan and Zueff had some concerns.^54 55^ As there were no studies with low levels of concern, subgroup analyses were not possible. Analysis using the DMPA trial arm from Beksinska^56^ was similar to that using the LNG-IUD arm (mean difference=−0.16 kg/m^2^; 95% CI: −0.52, 0.21; p=0.390; I^2^=75%; Egger’s test p value=0.0723; GRADE=very low) (online supplemental figure 2b).

#### Hormonal contraceptive versus non-hormonal contraceptives, non-randomised controlled trials (RCTs) and cohort studies

The evidence from non-randomised trials and cohort studies is very uncertain about the effect of the use of hormonal contraceptives compared with non-hormonal contraceptives on BMI. Five non-randomised trials plus three cohort studies compared hormonal contraceptives versus non-hormonal contraceptives on the outcome of BMI; however, one study^59^ was excluded from meta-analyses because the model failed to converge when included, due to imbalance in sample size. All seven included studies reported unadjusted estimates of BMI; however, BMI was balanced between all groups at baseline.

Two of the studies, Cursino and Quintino-Moro, compared the DMPA injection to the copper IUD.^60 61^ Four studies compared oral contraceptive pills to non-hormonal contraceptive methods (male condom or copper IUD): de Morais (ethinylestradiol/drospinonone),^44^ Nisenbaum (ethinylestradiol/drospinonone),^62^ Giribela (ethinylestradiol/drospinonone),^63^ and Franceschini (ethinylestradiol/chlormadinone acetate and ethinylestradiol/levonogestrel).^64^ As there were two intervention arms of relevance in the Franceschini study, two separate meta-analyses were carried out including either of these arms. Last, Oderich compared the etonogestrel implant to the copper IUD.^65^

Cursino and de Morais reported results per protocol,^44 60^ while the remaining studies reported intention-to-treat results. Cursino^60^ had serious risk of bias, while all others had critical risk of bias, per the ROBINS-I tool for analysis of risk of bias in non-randomised interventional studies. Using the ROBINS-E tool, Giribela^63^ was found to be of very high risk of bias. No subgroup analyses were performed as none were of low risk.

In both random-effects meta-analyses, no meaningful relationship was observed between BMI among those using hormonal contraceptives compared with those using non-hormonal contraceptives. For the meta-analysis using the ethinylestradiol/levonogestrel intervention arm of the Franchescini study,^64^ BMI was 0.05 kg/m^2^ higher in the hormonal contraceptive arm compared with non-hormonal contraception (mean difference=0.05 kg/m^2^; 95% CI: −0.16, 0.25; p=0.650; I^2^=0.0%; Egger’s test=0.734; GRADE=low) (online supplemental figure 3a). For that using the ethinylestradiol/chlormadinone intervention arm, BMI was 0.08 kg/m^2^ higher in the hormonal contraceptive arm compared with non-hormonal contraception (mean difference=0.08 kg/m^2^; 95% CI: −0.13, 0.28; p=0.450; I^2^=0.0%; Egger’s test=0.934; GRADE=low) (online supplemental figure 3b).

#### Hormonal contraceptives versus non-contraceptives

In addition to the trials included in meta-analyses, two non-randomised trials compared hormonal contraceptives to non-contraceptive interventions and found no difference in BMI between the groups.^66 67^

### Effects of contraceptive use on weight gain or loss

#### Hormonal contraceptive versus non-hormonal contraceptive, randomised controlled trials

The evidence suggests that using a hormonal contraceptive compared with a non-hormonal contraceptive may result in little to no difference in risk of self-reported weight loss or weight gain. Three studies compared hormonal contraceptives to non-hormonal contraceptives with the outcomes of weight gain and weight loss: Beesham, Haddad and Todd.^41 68 69^ Weight gain and weight loss were self-reported dichotomous outcomes for all studies (yes or no), and all studies reported intention-to-treat analyses. The studies reported only unadjusted results. All studies had a copper IUD as the non-hormonal contraceptive. Haddad compared this to the DMPA injection,^69^ Beesham compared with both the DMPA injection and the LNG-IUD^68^ and Todd compared with the LNG-IUD.^41^

Separate meta-analyses were conducted using either of the arms of the Beesham’s study,^68^ and both showed no effect of hormonal versus non-hormonal contraceptive use on weight gain (LNG-IUD arm: OR=1.09; 95% CI: 0.69, 1.73; p=0.710; I^2^=0%; Egger’s test p value=0.335; GRADE=low) (online supplemental figure 4a) (DMPA arm: OR=1.13; 95% CI: 0.72, 1.79; p=0.590; I^2^=0%; Egger’s test p value=0.105; GRADE=low) (online supplemental figure 4b). Similarly, no effects of hormonal vs non-hormonal contraceptive were observed for weight loss (LNG-IUD: OR=0.63; 95% CI: 0.37, 1.09; p=0.100; I2=0%; Egger’s test p value=0.425; GRADE=low) (online supplemental figure 5a) (DMPA: OR=0.59; 95% CI: 0.34, 1.02; p=0.060; I2=0%; Egger’s test p value=0.936; GRADE=low) (figure 5b). Both Haddad and Todd^41 69^ were found to be at high risk of bias, while Beesham^68^ was found to have some concerns.

Online supplemental tables 8–10 show details of GRADE assessments for each of the meta-analyses conducted.

### Effects of contraceptives on other anthropometric outcomes: narrative summary

Several studies reported on the outcomes of abdominal circumference, fat and lean mass and waist-hip ratios, though we were not able to conduct meta-analyses with these outcomes due to lack of sufficient comparability between interventions and outcomes. Three non-randomised studies reported on changes in abdominal circumference with non-hormonal contraceptives or no contraceptives versus oral contraceptives,^44 62 70^ and none found significant differences between the groups. An additional three non-randomised trials investigated changes in fat and lean mass with use of hormonal versus non-hormonal contraceptives.^60 61 70^ The only significant difference found in fat or lean mass among the study groups in these papers was in the Quintino Moro study, where the authors found an increase in fat mass in the hormonal contraceptive group (DMPA injection) compared with those using the copper IUD.^61^ Nine randomised controlled trials had waist-hip ratios as the outcome; four compared hormonal contraceptives to non-contraceptive interventions,^49 50 54 55^ and five compared different oral contraceptives to each other.^71–75^ None of the nine studies found any significant differences in the study groups with respect to waist-hip-ratios.

## Discussion

This systematic review and meta-analysis examined the effects of FP on nutritional outcomes among adolescent girls and WRA in LMICs. Despite limited evidence, meta-analyses indicated that women using hormonal IUDs had higher haemoglobin compared with those using oral contraceptives and those using hormonal contraceptives had slightly lower BMI than non-users. The quality of evidence is very uncertain, and no other associations of note were found. Null results for many of the meta-analyses may indicate limited impact of FP on some maternal nutrition outcomes; however, better quality evidence is needed before solidifying this conclusion.

Low haemoglobin levels are a strong predictor of adverse maternal and infant health outcomes, including low birth weight, preterm birth, perinatal mortality and neonatal mortality, among others.^76^ Contraceptives that reduce anaemia can help with recuperation and replenishment prior to or in between pregnancies, reducing maternal depletion syndrome and allowing for optimal health outcomes when pregnancy does occur.^76^ Hormonal contraceptives can decrease menstruation and limit blood loss compared with non-hormonal contraceptives. The relationship between hormonal IUD use and higher haemoglobin compared with use of oral contraceptives in our analyses shows that hormonal IUDs may be more effective than oral contraceptives in reducing menstrual blood losses. The progesterone-only contraceptive pill increases bleeding in-between menstrual cycles.^77^ Among the studies included in our meta-analyses, two used progesterone-only pills,^36 37^ and two used combined oral contraceptive pills.^38 39^ Given our results showing higher haemoglobin among users of hormonal IUDs compared with both progesterone-only and combined oral contraceptive pills, there may be an advantage to using the hormonal IUD among women at high risk of anaemia. The hormonal IUD may also be favourable to the copper IUD regarding its effects on haemoglobin levels. In a few studies not included in meta-analyses, but discussed in the narrative synthesis, use of LNG-IUD was associated with higher haemoglobin compared with the copper IUD.^40–42^ Further, hormonal IUD was associated with increased ferritin and haematocrit.^38 39^ However, data collection methods for haemoglobin were often not reported and may influence the reliability of the results.

Our findings align with existing research on the relationships between hormonal contraception and haemoglobin/anaemia levels. A previous systematic review not restricted to LMICs demonstrated that the LNG-IUD was associated with a significant increase in haemoglobin levels, compared with the copper IUD which resulted in a decrease in haemoglobin, although results of that review were also uncertain.^78^ In a study of 16 countries in Sub-Saharan Africa, as well as a study based only in Tanzania, use of hormonal contraceptives was associated with reduced likelihood of anaemia.^79 80^

Given the weak evidence base for the use of hormonal IUDs to reduce anaemia in LMICs, further implementation research is warranted to establish a stronger evidence base. Such research should carefully consider the barriers to adoption of hormonal IUDs in LMICs, including high costs, accessibility, potential side effects and cultural norms.

The slight reduction in BMI with hormonal contraception compared with no contraception is supported by mostly low risk of bias studies. However, no significant BMI differences were found between hormonal and non-hormonal contraceptives. Meta-analyses on BMI outcomes were highly heterogeneous and mostly involved populations with PCOS, a condition associated with chronic anovulation and hyperandrogenism, for which hormonal contraceptives are often employed to relieve symptoms.^81^ This may explain the observed BMI reduction with use of hormonal contraception, as higher BMI is a common symptom of PCOS.^82^ Treatment with hormonal contraceptives may thus reduce the symptom of increased BMI. Whether this reflects a true effect or is an artefact of poor evidence quality, the mechanisms underpinning lower BMI after hormonal contraceptive use remain unclear and interpretation is limited by the available studies.

Contrary to our findings of lower BMI after hormonal contraceptive use, weight gain is often expressed as a concern when considering oral contraceptives as an FP method in higher income countries.^83^ However, we found no evidence that weight loss or gain was significantly associated with any contraceptive use. In addition, no studies demonstrated differences in abdominal circumference, waist-hip ratio, fat mass or lean mass in relation to varying use of modern contraceptive methods. Of note, these outcomes are challenging to analyse without high risk of bias due to endogeneity, as they were collected as adverse effects or reasons for discontinuation of contraceptive use in the available studies. There is significant uncertainty regarding the influence of hormonal contraceptives on BMI and body composition, and further RCTs may be of benefit to elucidate the true relationship.

Overall, we found a lack of appropriate evidence to allow for strong and confident answers to our research questions about the relationships between use of FP and maternal nutrition outcomes. For many of our outcomes of interest, we were unable to conduct comparisons of contraceptive use to non-use of contraceptives due to the available study designs and data. Further, our intention was to investigate how FP exposure and interventions may change nutritional outcomes by reduction of maternal depletion through the pathways of delayed pregnancy, increased inter-pregnancy intervals, reduced parity and increased family income. However, among the included studies, none had long-term follow-up periods through multiple stages of reproductive life (eg, pre-pregnancy, pregnancy, post-partum, lactation). We are thus unable to draw conclusions regarding these longer-term mechanisms through which FP exposure or intervention is potentially related to nutritional outcomes. We are limited to more immediate pathways linked to changes to menstruation through contraceptive use. Similarly, we are unable to draw conclusions about how the duration of contraceptive use may be related to nutritional outcomes. In addition, nutrition-related outcomes were mostly reported as side effects of contraceptive use, including as reasons for discontinuation of use. As such, there is a high potential for selection bias due to differential lost to follow-up and confounding due to endogeneity in many of the available studies.

This work has several limitations beyond the paucity of appropriate evidence that should be considered in the interpretation of the results. The significant heterogeneity in intervention arms as well as outcomes reported restricted the possibilities for meta-analyses and makes it challenging to formulate strong, generalisable conclusions. Though 99 studies were included in the review and extracted, only six meta-analyses were possible and relevant, the largest of which included only seven studies. We intended to conduct several sub-analyses focusing on specific groups of interest such as adolescents; however, available data did not support these analyses. In addition, few studies had low risk of bias, further reducing the reliability of the results. We cannot rule out the potential for confounding in the observational studies, or selection bias in RCTs, as those who have access to contraceptives or to studies involving contraceptives may have general life circumstances more conducive to better nutritional status than those without access. However, many of the studies in the review compared one type of contraception to another type, in which case this type of confounding or selection bias is not relevant. Finally, control groups were not well defined in that there was often no mention of traditional methods such as the rhythm method, or barrier methods such as male or female condoms. It is impossible and unethical to prevent WRA from using any form of contraceptive, although traditional or modern, making it difficult to conduct research using strict control arms.

### Conclusion

Our systematic review found limited robust evidence on the relationships between FP and maternal nutrition outcomes. Weak evidence suggests hormonal FP methods may increase haemoglobin levels and decrease BMI. The lack of strong evidence highlights the need to establish a strong research agenda on relationships between FP interventions and maternal nutrition (and intermediate outcomes). Randomised controlled trials or cohort studies should focus on WRA and adolescents in LMICs. Such studies should also compare effectiveness of FP interventions to traditional anaemia programmes, including analysis of the risks of FP interventions compared with other strategies. Cost-effectiveness studies should be conducted to determine how cost-effective FP interventions are at addressing maternal nutrition compared with other strategies. Additionally, research on improving access to FP for vulnerable populations such as adolescents is needed. Further investigations are critical to determine the impact of modern contraceptive methods on maternal nutrition outcomes in LMICs, to support the integration of FP and nutrition interventions.

## Data Availability

Data are available upon reasonable request. All data referenced in the article were pulled from existing articles. Extracted data are available upon request from the authors, as is the original protocol.
