# Supplementary material for: Family planning and nutrition: systematic review of the effects of family planning on nutritional status of adolescent girls and women of reproductive age
Source: BMJ Glob Health. 2025 Apr 25;10(Suppl 1):e015734. doi: 10.1136/bmjgh-2024-015734 (PMC12184413; doi:10.1136/bmjgh-2024-015734)
Supplement: Supplementary data [file bmjgh-10-Suppl_1-s001.pdf]

**Supplementary material for: Family planning and nutrition: systematic review of the effects of family planning and related practices on nutritional status of adolescent girls and women of reproductive age**

**Supplementary Table 1: Search Strategy: Systematic review of effect of family planning on nutrition**

| No. | Concept | Search terms | PubMed records (February 28th, 2023) | Web of Science records (February 24, 2023) | Embase records (February 20, 2023) | Cochrane records (February 24, 2023) |
|---|---|---|---|---|---|---|
| **Family Planning** | | | | | | |
| #1 | Family planning | contracepti*[tiab] OR family planning[tiab] OR family planning[mh] OR birth control[tiab] OR birth control[mh] OR contraception injectable*[tiab] OR IUD[tiab] OR IUCD[tiab] OR IUS OR intrauterine device*[tiab] OR intra-uterine device*[tiab] OR intrauterine system*[tiab] OR intra-uterine system*[tiab] OR oral contraceptive pill*[tiab] OR hormonal contraceptive pill*[tiab] OR birth control pill[tiab] OR emergency contraceptive pill*[tiab] OR cervical cap*[tiab] OR vaginal diaphragm*[tiab] OR vaginal ring*[tiab] OR implant*[tiab] OR subdermal implant*[tiab] OR implanon[tiab] OR jadelle[tiab] OR norplant*[tiab] OR sino-implant*[tiab] OR sinoimplant*[tiab] OR injectable*[tiab] OR contraceptive patch[tiab] OR sterilization[tiab] OR sterilisation[tiab] OR vasectomy[tiab] OR contraception behaviour[tiab] OR long-acting reversible contraception[tiab] OR family planning[mh] OR birth control[mh] OR contraception[mh] OR Family Planning Services[mh] OR "sterilization, reproductive"[MeSH Terms] OR lactational amenorrhea method [tiab] OR lactational amenorrhoea method[tiab] OR sympto-thermal method[tiab] OR twoday method[tiab] OR standard days method[tiab] OR basal body temperature method[tiab] OR rhythm method[tiab] OR calendar method[tiab] OR condom*[tiab] OR withdrawal[tiab] OR coitus interruptus[tiab] | 742,286 | 878,222 | 363,608 | 133,142 |
| #2 | Induced abortion | “Abortion, induced”[Mesh] OR Induced Abortion*[tiab] OR Drug-induced Abortion* OR illegal Abortion*[tiab] OR criminal Abortion*[tiab] OR unsafe Abortion*[tiab] OR induced termination of pregnancy[tiab] OR illegal termination of pregnancy[tiab] OR criminal termination of pregnancy[tiab] OR unsafe termination of pregnancy[tiab] OR medical termination of pregnancy[tiab] | 46,597 | 3,780,312 | 11,860 | 3,299 |
| **Nutritional Status** | | | | | | |
| #3 nutritional status | | "Nutritional Status"[Mesh] OR nutrition[tiab] OR "Overnutrition"[Mesh] OR overnutrition[tiab] OR “over-nutrition”[tiab] OR "Malnutrition"[Mesh] OR malnutrition[tiab] OR malnourished[tiab] OR undernutrition[tiab] OR undernourished[tiab] OR "Hemoglobins"[Mesh] OR hemoglobin*[tiab] OR haemoglobin*[tiab] OR "Anemia"[Mesh] OR anemia[tiab] OR anaemia[tiab] OR anemic[tiab] OR anaemic[tiab] | 1,047,814 | 728,015 | 1,995,304 | 114,411 |

| | | | | | |
|---|---|---|---|---|---|
| #4 Micronutrients | "Micronutrients"[Mesh] OR micronutrient*[tiab] OR "Minerals"[Mesh] OR Mineral*[tiab] OR "Vitamins"[Mesh] OR vitamin*[tiab] OR "Dietary Supplements"[Mesh] OR "Calcium"[Mesh] OR calcium[tiab] OR "Magnesium"[Mesh] OR magnesium[tiab] OR "Phosphorus, Dietary"[Mesh] OR phosphorus[tiab] OR "Potassium, Dietary"[Mesh] OR potassium[tiab] OR "Boron"[Mesh] OR boron[tiab] OR "Cobalt"[Mesh] OR cobalt[tiab] OR "Chromium"[Mesh] OR chromium[tiab] OR "Copper"[Mesh] OR copper[tiab] OR "Iodine"[Mesh] OR iodine[tiab] OR "Iron"[Mesh] OR iron[tiab] OR "Ferritins"[Mesh] OR ferritin[tiab] OR "Transferrin"[Mesh] OR transferrin[tiab] OR "Hematocrit"[Mesh] OR hematocrit[tiab] OR haematocrit[tiab] OR "Manganese"[Mesh] OR manganese[tiab] OR "Molybdenum"[Mesh] OR molybdenum[tiab] OR "Selenium"[Mesh] OR selenium[tiab] OR "Zinc"[Mesh] OR zinc[tiab] OR "Thiamine"[Mesh] OR thiamin*[tiab] OR "Riboflavin"[Mesh] OR riboflavin[tiab] OR "Niacin"[Mesh] OR niacin[tiab] OR "Pantothenic Acid"[Mesh] OR "pantothenic acid"[tiab] OR "Pyridoxine"[Mesh] OR pyridox*[tiab] OR "Biotin"[Mesh] OR biotin[tiab] OR "Folic Acid"[Mesh] OR folate[tiab] OR "folic acid"[tiab] OR "Vitamin B 12"[Mesh] OR cobalamin*[tiab] OR "Methylmalonic Acid"[Mesh] OR "Methylmalonic acid*"[tiab] OR "Homocysteine"[Mesh] OR homocysteine[tiab] OR "Vitamin A"[Mesh] OR retinol[tiab] OR "Ascorbic Acid"[Mesh] OR "ascorbic acid"[tiab] OR calciferol*[tiab] OR "Ergocalciferols"[Mesh] OR ergocalciferol[tiab] OR "Vitamin D"[Mesh] OR cholecaliferol[tiab] OR "Tocopherols"[Mesh] OR tocopherol*[tiab] OR "Tocotrienols"[Mesh] OR tocotrienol*[tiab] OR "Vitamin K"[Mesh] OR phylloquinone[tiab] OR menaquinone[tiab] OR "Choline"[Mesh] OR Choline[tiab] OR "Carotenoids"[Mesh] OR carotenoid*[tiab] OR carotene[tiab] | 2,470,065 | 4,438,725 | 3,915,314 | 147,131 |
| #5 Macronutrients | "Nutrients"[Mesh] OR nutrient*[tiab] OR macronutrient*[tiab] OR "Carbohydrates"[Mesh] OR carbohydrate*[tiab] OR "Glucose"[Mesh] OR glucose[tiab] OR "Dietary Fiber"[Mesh] OR fiber*[tiab] OR fat[tiab] OR "Fatty Acids"[Mesh] OR "fatty acid*"[tiab] OR "Omega-3"[tiab] OR "Omega-6"[tiab] OR "Lipids"[Mesh] OR lipid*[tiab] OR "Triglycerides"[Mesh] OR triglyceride*[tiab] OR "Cholesterol"[Mesh] OR cholesterol*[tiab] OR "Proteins"[Mesh] OR protein*[tiab] OR "Amino Acids"[Mesh] OR "amino acid*"[tiab] | 10,650,493 | 7,300,629 | 321,786 | 401,068 |
| #6 Body composition | "Body Composition"[Mesh] OR "body compostion*"[tiab] OR "fat-to-lean mass ratio"[tiab] OR "total body water"[tiab] OR "doubly labelled water"[tiab] OR "deuterium water"[tiab] OR "bioelectrical impedance analysis"[tiab] | 67,555 | 86,354 | 128,860 | 7,483 |
| #7 Anthropometry | "Body Weight"[Mesh] OR Weight[tiab] OR "Body Mass Index"[Mesh] OR "body mass index"[tiab] OR BMI[tiab] OR "Obesity"[Mesh] OR obesity[tiab] OR obese[tiab] OR "Thinness"[Mesh] OR underweight[tiab] OR "Weight Gain"[Mesh] OR "Body Weight Changes"[Mesh] OR "Body-Weight Trajectory"[Mesh] OR "Overweight"[Mesh] OR overweight[tiab] OR "Anthropometry"[Mesh] OR anthropometry[tiab] OR anthropometric[tiab] OR height OR stature OR stunting[tiab] OR stunted[tiab] OR "mid-upper arm circumference"[tiab] OR | 1,847,886 | 3,359,296 | 854,724 | 210,857 |

| | | | | | |
|---|---|---|---|---|---|
| | “waist circumference”[tiab] OR “BMI-for-age”[tiab] OR “weight-for-age”[tiab] OR “height-for-age”[tiab] | | | | |
| #8 Gestational Weight Gain | "Gestational Weight Gain"[Mesh] OR “Weight Gain, Gestational”[tiab] OR “Pregnancy Weight Gain”[tiab] OR “Weight Gain, Pregnancy”[tiab] OR “Maternal Weight Gain”[tiab] OR “Weight Gain, Maternal”[tiab] OR “Postpartum Weight Retention”[tiab] OR “Weight Retention, Postpartum”[tiab] | 3,481 | 100,038 | 7,020 | 594 |
| **Population** | | | | | |
| #9 Adolescents and women of reproductive age | "Women"[Mesh] OR women[tiab] OR woman[tiab] OR "Adolescent"[Mesh] OR Adolescent*[tiab] OR teen[tiab] OR teens[tiab] OR teenager*[tiab] OR girl*[tiab] OR "Mothers"[Mesh] OR mother*[tiab] | 3,720,166 | 2,522,299 | 12,478,834 | 333,690 |
| #10 Low- and middle-income countries | "Developing Countries"[MeSH] OR “developing countr*”[tiab] OR “developing nation*”[tiab] OR “less developed countr*”[tiab] OR “less developed nation*”[tiab] OR “third world nation*”[tiab] OR “third world countr*”[tiab] OR “under developed nation*”[tiab] OR “underdeveloped nation*”[tiab] OR “under developed countr*”[tiab] OR “underdeveloped countr*”[tiab] OR “middle income countr*”[tiab] OR “middle-income countr*”[tiab] OR “middle income nation*”[tiab] OR “middle-income nation*”[tiab] OR “low income countr*”[tiab] OR “low-income countr*”[tiab] OR “low income nation*”[tiab] OR “low-income nation*”[tiab] OR “poor countr*”[tiab] OR “poor nation*”[tiab] OR LMIC[tiab] OR LMICs[tiab] OR "Africa"[MeSH] OR "Asia"[MeSH] OR "South America"[MeSH] OR "Latin America"[MeSH] OR "Central America"[MeSH] OR africa[tiab] OR asia[tiab] OR “south america*”[tiab] OR “latin america*”[tiab] OR “central america*”[tiab] OR Afghanistan*[tiab] OR Albania*[tiab] OR Algeria*[tiab] OR Samoa*[tiab] OR Angola*[tiab] OR Armenia*[tiab] OR Azerbaijan*[tiab] OR Bangladesh*[tiab] OR Bengali[tiab] OR Belarus*[tiab] OR Belize[tiab] OR Benin[tiab] OR Bhutan*[tiab] OR Bolivia*[tiab] OR Bosnia*[tiab] OR Herzegovina*[tiab] OR Botswana*[tiab] OR Brazil*[tiab] OR Bulgaria*[tiab] OR “Burkina Faso”[tiab] OR Burkinabe[tiab] OR Burundi*[tiab] OR “Cabo Verd*”[tiab] OR “Cape Verd*”[tiab] OR Cambodia*[tiab] OR Cameroon*[tiab] OR “Central African*”[tiab] OR Chad*[tiab] OR China[tiab] OR Chinese[tiab] OR Colombia*[tiab] OR Comoros[tiab] OR Congo[tiab] OR “Costa Rica*”[tiab] OR “Cote d'Ivoire”[tiab] OR “Ivory Coast”[tiab] OR Cuba[tiab] OR Cuban[tiab] OR Djibouti[tiab] OR Dominica*[tiab] OR Ecuador[tiab] OR Egypt*[tiab] OR “El Salvador*”[tiab] OR Eritrea*[tiab] OR Ethiopia*[tiab] OR Fiji*[tiab] OR Gabon*[tiab] OR Gambia*[tiab] OR Georgia*[tiab] OR Ghana*[tiab] OR Grenada*[tiab] OR Guatemala*[tiab] OR Guinea*[tiab] OR Guyan*[tiab] OR Haiti*[tiab] OR Hondura*[tiab] OR India[tiab] OR Indian*[tiab] OR Indonesia*[tiab] OR Iran*[tiab] OR Iraq*[tiab] OR Jamaica*[tiab] OR Jordan*[tiab] OR Kazakh*[tiab] OR Kenya*[tiab] OR Kiribati[tiab] OR “People's Republic of Korea”[tiab] OR “North Korea”[tiab] OR Kosovo[tiab] OR Kosovar*[tiab] OR Kyrgyz*[tiab] OR Lao[tiab] OR Laos[tiab] OR Laotian*[tiab] OR Lebanon[tiab] OR Lebanes*[tiab] OR Lesotho[tiab] OR Liberia*[tiab] OR Libya*[tiab] OR | 2,642,185 | 5,412,598 | 3,415,222 | 1,134,485 |

| | | | | | |
|---|---|---|---|---|---|
| | Macedonia*[tiab] OR Madagascar*[tiab] OR Malawi*[tiab] OR Malaysia*[tiab] OR Maldives[tiab] OR Mali[tiab] OR "Marshall Island*"[tiab] OR "Mexico"[MeSH] OR Mexico[tiab] OR Mexican*[tiab] OR Micronesia*[tiab] OR Moldova*[tiab] OR Mongolia*[tiab] OR Montenegr*[tiab] OR Morocc*[tiab] OR Mozambique[tiab] OR Myanmar[tiab] OR Burmese*[tiab] OR Burma[tiab] OR Namibia*[tiab] OR Nepal*[tiab] OR Nicaragua*[tiab] OR Niger*[tiab] OR Pakistan*[tiab] OR Paraguay*[tiab] OR Peru*[tiab] OR Philippin*[tiab] OR Rwanda*[tiab] OR "Sao Tome"[tiab] OR Principe[tiab] OR Senegal*[tiab] OR Serbia*[tiab] OR "Sierra Leone*"[tiab] OR "Solomon Island*"[tiab] OR Somalia*[tiab] OR "South Africa*"[tiab] OR "Sri Lanka"[tiab] OR "St Lucia"[tiab] OR "Saint Lucia"[tiab] OR "St Vincent"[tiab] OR "Saint Vincent"[tiab] OR Grenad*[tiab] OR Sudan*[tiab] OR Suriname*[tiab] OR Swaziland*[tiab] OR Eswatini*[tiab] OR Syria*[tiab] OR Tajik*[tiab] OR Tanzania*[tiab] OR Zanzibar[tiab] OR Thai*[tiab] OR Timor*[tiab] OR Togo*[tiab] OR Tonga*[tiab] OR Tunisia*[tiab] OR Turkey[tiab] OR Turkish[tiab] OR Turkmen*[tiab] OR Tuvalu*[tiab] OR Uganda*[tiab] OR Ukrain*[tiab] OR Uzbeki*[tiab] OR Vanuatu*[tiab] OR Venezuela*[tiab] OR Vietnam*[tiab] OR "Viet nam*"[tiab] OR "West Bank"[tiab] OR Gaza*[tiab] OR Palestin*[tiab] OR Yemen*[tiab] OR Zambia*[tiab] OR Zimbabw*[tiab] OR "Western Sahara"[tiab] OR Argentin*[tiab] OR Russia*[tiab] OR Maurit*[tiab] OR Palau[tiab] | | | | |
| COMBINATIONS | | | | | |
| Family planning AND Nutrition Status of Adolescent and women in LMIC | | | | | |
| | (#1) AND (#3 OR #4 OR #5 OR #6 OR #7 OR #8) AND (#9) AND (#10) | 5,647 | 2,466 | 3,755 | 13,577 |
| Induced Abortion AND Nutrition Status in Adolescent and women in LMIC | | | | | |
| | (#2) AND (#3 OR #4 OR #5 OR #6 OR #7 OR #8) AND (#9) AND (#10) | 865 | 3,168 | 3,251 | 806 |
| Family Planning OR Induced Abortion AND Nutrition Status of Adolescent and women in LMIC | | | | | |
| | (#1 OR #2) AND (#3 OR #4 OR #5 OR #6 OR #7 OR #8) AND (#9) AND (#10) | 6,152 | 5,391 | | 14,204 |
| Family Planning OR Induced Abortion AND Nutrition Status of Adolescent and women in LMIC restricted by year | | | | | |

| | | | | | |
|---|---|---|---|---|---|
| | (#1 OR #2) AND (#3 OR #4 OR #5 OR #6 OR #7 OR #8) AND (#9) AND (#10) after 2000 | 3,518 | 4,957 | | 12,967 |
| Family Planning OR Induced Abortion AND Nutrition Status of Adolescent and women in LMIC restricted by year and English language | | | | | |
| | (#1 OR #2) AND (#3 OR #4 OR #5 OR #6 OR #7 OR #8) AND (#9) AND (#10) after 2000 and English language only | 3,351 | 4,860 | | 12,815 |

**Supplementary Table 2: List of data extracted for review of family planning and nutrition among women of reproductive age**

| **Descriptive** | **Categorical** | **Continuous** |
|---|---|---|
| Paper, date | Paper, date | Paper, date |
| First author | Categorical outcome | Continuous outcome |
| Corresponding author | Intervention arm | Intention to treat or per protocol |
| Corresponding author institution | Intention to treat or per protocol | Continuous outcome unit |
| Corresponding author email | Categorization | Intervention arm |
| Journal | Intervention group total sample size | Intervention group sample size pre |
| Country | Intervention success sample size | Intervention group mean pre |
| Rural or urban | Intervention failure sample size | Intervention group standard error pre |
| Study registration | Control group total sample size | Intervention group sample size post |
| Registration type | Control success sample size | Intervention group mean post |
| Trial funding | Control failure sample size | Intervention group standard error post |
| Trial grant | Adjusted or unadjusted results | Intervention sample size difference |
| Study design | Results ratio | Difference in intervention means pre/post |
| Study period | Ratio estimate | Mean difference intervention pre/post |
| Study population | Standard error | Difference in intervention SE pre/post |
| Participant selection | Lower CI | SE difference intervention pre/post |
| Sample randomization | Upper CI | Control sample size pre |
| Inclusion criteria | Bias tool | Control mean pre |
| Exclusion criteria | | Control SE pre |
| Eligible age group | | Control sample size post |
| Age type | | Control mean post |
| Average age | | Control SE post |
| Average age intervention 1-intervention 6 | | Control sample size difference |
| Average age control | | Difference in control means pre/post |
| Age standard deviation/iqr | | Mean difference control pre/post |
| Age standard deviation/iqr intervention 1 – intervention 6 | | Difference in SE control pre/post |
| Age standard deviation/iqr control | | SE difference control pre/post |

| | | |
|---|---|---|
| Intervention 1 – intervention 6 | | Adjusted results |
| Intervention 1 time point (pregnant, non-pregnant, postpartum, etc.) – intervention 6 time point | | Estimate |
| Intervention 1 – 6 specifications | | Standard error |
| Intervention 1 – 6 doses | | Lower CI |
| Intervention 1 – 6 frequencies | | Upper CI |
| Intervention 1 – 6 durations | | Values reported (mean, SD, SE, etc.) |
| Control | | Bias tool |
| Control timepoint | | |
| Control specification | | |
| Control dose | | |
| Control frequency | | |
| Control duration | | |
| Cointervention | | |
| Outcome 1 – outcome 7 | | |
| Outcome 1-7 types (continuous, categorical) | | |
| Outcome 1-7 timepoints | | |
| Outcome 1-7 units | | |
| Outcome 1-7 ascertained | | |
| Outcome 1-7 categories | | |
| Outcome 1-7 remarks | | |
| Allocation concealment | | |
| Participant blinding | | |
| Staff blinding | | |
| Analyst blinding | | |
| Analysis method | | |
| Intention to treat or per protocol | | |
| Number of clusters | | |
| Intra cluster correlation coefficient | | |

| | | |
|---|---|---|
| Sample size enrolled | | |
| Sample size protocol | | |
| Sample size follow up | | |
| Sample size baseline intervention 1-6 | | |
| Sample size follow-up intervention 1-6 | | |
| Sample size baseline control | | |
| Sample size follow-up control | | |
| Sample size lost to follow up | | |
| Notes | | |

**Supplementary Table 3. Characteristics of studies extracted for review of family planning and nutrition among women of reproductive age**

| Study | Country | Population | Sample Size | Study Design | Arms | Outcomes |
|---|---|---|---|---|---|---|
| Abu Hashim, 2012 | Egypt | Women of reproductive age with heavy menstrual bleeding | 95 | RCT | Oral contraceptive<br>Vaginal ring | Ferritin<br>Hemoglobin |
| Afsar, 2005 | Pakistan | Women of reproductive age | 50 | Non-randomized trial | Control (no contraception)<br>Injectable | BMI<br>Hematocrit<br>Hemoglobin %<br>Weight |
| Al-Ghashri, 2021 | Oman | Women of reproductive age | 404 | Retrospective cohort | Oral contraceptive x 2<br>Injectable | Weight gain |
| Amiri, 2020a | Iran | Women of reproductive age with PCOS | 120 | RCT | Oral contraceptive x 4 | BMI<br>Central obesity<br>Obesity<br>Visceral adiposity<br>Waist circumference<br>Waist-height ratio<br>Waist-hip-ratio |
| Amiri, 2020b | Iran | Women of reproductive age with PCOS | 200 | RCT | Oral contraceptive x 6 | BMI<br>Waist circumference<br>Waist-hip ratio<br>Weight |
| Andrade, 2004 | Brazil | Women of reproductive age | 70 | Non-randomized trial | Hormonal IUD<br>Non-hormonal IUD x 2 | Ferritin |
| Arowojolu, 2003 | Nigeria | Women of reproductive age | 462 | Prospective cohort | Implant x 2 | Weight<br>Weight gain<br>Weight loss |

| Study | Country | Population | Sample Size | Study Design | Arms | Outcomes |
|---|---|---|---|---|---|---|
| Barham, 2021 | Bangladesh | Women of reproductive age | 59,189 | Retrospective cohort | In-home delivery of contraceptives with counseling<br>Clinic provision of contraceptives | BMI |
| Beesham, 2022 | South Africa | Women and adolescents of reproductive age | 829 | RCT | Implant<br>Injectable<br>Non-hormonal IUD | Weight gain<br>Weight loss |
| Behboudi-Gandevani, 2019 | Iran | Women of reproductive age with PCOS | 64 | RCT | No contraception (Phlebotomy)<br>Oral contraceptive | BMI<br>Waist-hip-ratio |
| Behnamfar, 2014 | Iran | Women with endometrial hyperplasia | 60 | RCT | Hormonal IUD<br>Oral contraceptive | Weight gain |
| Beksinska, 2021 | Eswatini<br>Kenya<br>South Africa<br>Zambia | Women and adolescents of reproductive age | 7,829 | RCT | Hormonal IUD<br>Injectable<br>Non-hormonal IUD | BMI<br>Weight |
| Bilghan, 2015 | Turkey | Women who underwent voluntary pregnancy termination | 100 | RCT | Hormonal IUD<br>Non-hormonal IUD | BMI<br>Hemoglobin<br>Waist-hip ratio<br>Weight |
| Brache, 2021 | Dominican Republic | Women of reproductive age | 49 | RCT | Non-hormonal IUD + Ulipristal Acetate x 4 different doses | Hematocrit<br>Hemoglobin |

| Study | Country | Population | Sample Size | Study Design | Arms | Outcomes |
|---|---|---|---|---|---|---|
| Braga, 2014 | Brazil | Women of reproductive age with thrombophilia and/or a history of venous thromboembolism (VTE) | 33 | Prospective cohort | Hormonal IUD<br>Hormonal IUD + Oral anticoagulant therapy (OAT) | Abdominal circumference<br>BMI<br>Hematocrit<br>Hemoglobin %<br>Weight |
| Bright, 2022 | Bangladesh | Postpartum women | 3,697 | Non-randomized trial | Control (no contraception)<br>Hormonal IUD | Hemoglobin |
| Brito, 2009 | Brazil | Women of reproductive age | 40 | RCT | Implant<br>Injectable | BMI<br>Hemoglobin<br>Waist circumference<br>Weight |
| Burke, 2018 | Malawi | Women of reproductive age | 731 | RCT | Injectable x 2 (self administered vs provider administered) | Weight change |
| Caldeirão, 2022 | Brazil | Adolescents | 127 | Non-randomized trial | Control (no contraception)<br>Oral contraceptive x 2 | BMI<br>BMI percentile<br>Fat/lean mass<br>Height<br>Total body fat<br>Weight |
| Carbonell, 2008 | Cuba | Pregnant women | 210 | RCT | Abortion drug x 2 | Hemoglobin |
| Chappell, 2017 | Uganda | Women of reproductive age with HIV | 60 | Non-randomized trial | Implant<br>Implant + antiretroviral therapy x 2 | Weight gain |

| Study | Country | Population | Sample Size | Study Design | Arms | Outcomes |
|---|---|---|---|---|---|---|
| Chappell, 2019 | Malawi, South Africa, Uganda and Zimbabwe | Women of reproductive age | 2,692 | Prospective cohort | Hormonal IUD<br>Implant<br>Injectable x 2<br>Oral contraceptive | Weight gain |
| Cursino, 2018 | Brazil | Women of reproductive age | 39 | Non-randomized trial | Injectable<br>Non-hormonal IUD | BMI<br>Fat/lean mass<br>Total body fat<br>Waist-hip ratio<br>Weight |
| Dabash, 2010 | Egypt | Women presenting with incomplete abortion | 697 | RCT | Abortion drug<br>Abortion surgical intervention | Hemoglobin |
| Dal'Ava, 2012 | Brazil | Women of reproductive age | 76 | Non-randomized trial | Hormonal IUD<br>Non-hormonal IUD | Central-peripheral fat ratio<br>Change in fat mass<br>Change in lean mass<br>Weight |
| Dal'Ava, 2014 | Brazil | Women of reproductive age | 97 | Prospective cohort | Control (no contraception)<br>Injectable<br>Non-hormonal IUD | Central-peripheral fat ratio<br>Fat/lean mass<br>Weight |
| de Morais, 2014 | Brazil | Women of reproductive age with hypertension | 60 | Non-randomized trial | Non-hormonal contraceptive<br>Oral contraceptive | Abdominal circumference<br>BMI<br>Hemoglobin<br>Weight |
| Deo, 2021 | India | Women and adolescents of reproductive age | 64 | RCT | Oral contraceptive<br>Oral contraceptive + non-contraceptive drug | BMI<br>Waist circumference<br>Waist-hip-ratio |

| Study | Country | Population | Sample Size | Study Design | Arms | Outcomes |
|---|---|---|---|---|---|---|
| | | | | | Supplementation (no contraception) | |
| dos Santos Quaresma, 2021 | Brazil | Women of reproductive age | 32 | Non-randomized trial | No hormonal contraceptive use<br>Oral contraceptive | Abdominal circumference<br>Fat/lean mass<br>Waist circumference<br>Weight |
| Erol, 2014 | Turkey | Women of reproductive age | 150 | RCT | Hormonal IUD<br>Non-hormonal IUD | Hemoglobin |
| Fekih, 2010 | Tunisia | Pregnant women | 252 | RCT | Abortion drug x 2 | Hematocrit<br>Hemoglobin |
| Franceschini, 2012 | Brazil | Women of reproductive age | 64 | Prospective cohort | Non-hormonal contraceptive<br>Oral contraceptive x 2 | BMI<br>Weight |
| Gallo, 2016 | Jamaica | Women of reproductive age | 414 | RCT | Control (no contraception)<br>Implant | Weight change<br>Weight gain<br>Weight loss |
| Giribela, 2012 | Brazil | Women of reproductive age | 71 | Prospective cohort | Non-hormonal contraceptive<br>Oral contraceptive | BMI<br>Weight |
| Gomes, 2012 | Brazil | Women of reproductive age with PCOS | 60 | Non-randomized trial | No contraception<br>Oral contraception x 2 | BMI<br>Waist circumference |
| Guang-Sheng, 2010 | China | Women of reproductive age | 768 | RCT | Oral contraceptive x 2 | Weight change<br>Weight gain |
| Haddad, 2013 | Malawi | Women of reproductive age, HIV positive | 200 | RCT | Injectable<br>Non-hormonal IUD | Weight gain<br>Weight loss |
| Hassanin, 2020 | Egypt | Women of reproductive age with symptomatic adenomyosis | 110 | RCT | Oral contraceptive x 2 | Weight gain |

| Study | Country | Population | Sample Size | Study Design | Arms | Outcomes |
|---|---|---|---|---|---|---|
| Hernandez-Juarez, 2014 | Mexico | Women of reproductive age | 100 | Prospective cohort | Implant<br>Skin patch | Abdominal circumference<br>BMI<br>Weight |
| Hou, 2010 | China | Women seeking voluntary pregnancy termination | 100 | RCT | Abortion drug x 2 | Hemoglobin |
| Hubacher, 2015 | Kenya | Postpartum women | 313 | Prospective cohort | Hormonal IUD<br>Implant | Weight gain<br>Weight loss |
| Ibrahim, 2019a | Iraq | Women with missed miscarriages | 120 | RCT | Abortion drug x 2 | Hemoglobin |
| Ibrahim, 2019b | Malaysia | Women and adolescents of reproductive age | 380 | Retrospective cohort | Control (no contraception)<br>Injection<br>Non-hormonal IUD<br>Oral contraceptive x 2 | Weight change |
| Ilyin, 2021 | Russia | Women of reproductive age with heavy menstrual bleeding | 312 | RCT | Hormonal IUD x 2 | BMI<br>Ferritin<br>Hemoglobin<br>Weight<br>Weight gain |
| Iwata, 2015 | Brazil | Women with PCOS | 41 | Non-randomized trial | Oral contraceptive<br>Oral contraceptive + non-contraceptive drug | BMI |
| Jamali, 2014 | Iran | Women of reproductive age | 80 | RCT | Injection<br>Oral contraceptive | Weight gain |
| Jimoh, 2021 | Nigeria | Women of reproductive age | 240 | Prospective cohort | Non-hormonal contraceptive<br>Oral contraceptive | BMI |
| Kachhawa, 2021 | India | Adolescents and young women of reproductive age | 70 | RCT | Oral contraceptive<br>Supplementation (no contraception) | BMI<br>Waist-hip-ratio<br>Weight |

| Study | Country | Population | Sample Size | Study Design | Arms | Outcomes |
|---|---|---|---|---|---|---|
| Kahraman, 2014 | Turkey | Women of reproductive age with PCOS | 52 | RCT | Oral contraceptive x 2 | % Change in BMI<br>% Change in waist-hip-ratio |
| Kakaire, 2015 | Uganda | Women of reproductive age with HIV | 703 | RCT | Hormonal IUD<br>Non-hormonal IUD | Weight change |
| Karimi-Zarchi, 2013 | Iran | Women of reproductive age with endometrial hyperplasia with abnormal uterine bleeding (AUB) | 40 | RCT | Hormonal IUD<br>Oral contraceptive | Weight gain |
| Kashanian, 2010 | Iran | Women and adolescents of reproductive age | 342 | RCT | Oral contraceptive x 2 | Weight gain<br>Weight loss |
| Kavasoglu, 2020 | Turkey | Women of reproductive age with heavy menstrual bleeding | 192 | RCT | Hormonal IUD<br>Oral contraceptive | Hemoglobin<br>Weight gain |
| Kelekci, 2012a | Turkey | Women of reproductive age | 74 | Non-randomized trial | Hormonal IUD x 2<br>Non-hormonal IUD | Hemoglobin |
| Kelekci, 2012b | Turkey | Women of reproductive age with hirutism | 166 | RCT | Oral contraceptive x 2<br>Oral contraceptive + non-contraceptive drug | Weight change |
| Kilic, 2011 | Turkey | Women of reproductive age with obesity and PCOS and impaired glucose tolerance | 105 | RCT | Non-contraceptive drug (metformin) x 2<br>Oral contraceptive x 2 | Abdominal circumference<br>BMI |
| Kumar, 2005 | India | Pregnant women seeking abortion | 90 | Retrospective cohort | Abortion drug x 2 | Hemoglobin |

| Study | Country | Population | Sample Size | Study Design | Arms | Outcomes |
|---|---|---|---|---|---|---|
| Kumar,2018 | India | Women of reproductive age with PCOS | 90 | RCT | Non-contraceptive drug (metformin)<br>Oral contraceptive<br>Oral contraceptive + non-contraceptive drug (metformin) | BMI<br>Percent android/gynoid fat<br>Total body fat<br>Weight |
| Machado, 2006 | Brazil | Women of reproductive age | 90 | RCT | Non-hormonal contraception<br>Oral contraceptive x 2 | Fat/lean mass<br>Weight |
| Malik, 2020 | Pakistan | Women of reproductive age | 76 | RCT | Hormonal IUD<br>Oral contraceptive | |
| Mawet, 2014 | Serbia, Romania, Macedonia | Women of reproductive age with heavy menstrual bleeding | 280 | RCT | Hormonal IUD x 2 | Ferritin<br>Hemoglobin |
| Mehrabian, 2016 | Iran | Women of reproductive age with PCOS and metabolic syndrome | 111 | RCT | Non-contraceptive drug x 2 (metformin, simvastatin)<br>Oral contraceptive | BMI<br>Waist circumference |
| Melhado-Kimura, 2018 | Brazil | Women of reproductive age | 48 | Non-randomized trial | Injectable<br>Non-hormonal IUD | BMI<br>Central-peripheral fat ratio<br>Fat/lean mass<br>Total mass<br>Weight |
| Mohamed, 2011 | Egypt | Women of reproductive age | 600 | RCT | Oral contraceptive<br>Vaginal ring | Weight gain |
| Momeni, 2020 | Iran | Women of reproductive age | 100 | Prospective cohort | Control (no contraception)<br>Oral contraceptive | BMI<br>Waist-hip ratio |

| Study | Country | Population | Sample Size | Study Design | Arms | Outcomes |
|---|---|---|---|---|---|---|
| Moreira, 2020 | Brazil | Women of reproductive age | 80 | Prospective cohort | Hormonal IUD<br>Implant<br>Non-hormonal IUD x 2 | Hemoglobin |
| Nakalema, 2022 | Uganda | Women of reproductive age with and without HIV | 81 | Non-randomized trial | Implant<br>Implant + antiretroviral therapy | Weight gain |
| Nisenbaum, 2014 | Brazil | Women of reproductive age | 80 | Non-randomized trial | Non-hormonal contraceptive<br>Oral contraceptive | Abdominal circumference<br>BMI |
| Nooh, 2016 | Egypt | Women of reproductive age with endometrial hyperplasia with abnormal uterine bleeding (AUB) | 158 | RCT | Injectable<br>Oral contraceptive | Weight gain |
| Oderich, 2012 | Brazil | Women of reproductive age | 40 | Non-randomized trial | Implant<br>Non-hormonal IUD | BMI |
| Ortayli, 2001 | Turkey | Women of reproductive age | 105 | Prospective cohort | Control (no contraception)<br>Hormonal IUD<br>Implant | Weight |
| Ozay, 2016 | Turkey | Women of reproductive age with PCOS | 137 | RCT | Oral contraceptive<br>Supplementation (myoinositol + folic acid) | BMI<br>Waist-hip ratio |
| Özdemir, 2008 | Turkey | Women of reproductive age with PCOS | 79 | RCT | Oral contraceptive x 2 | BMI<br>Waist-hip ratio |
| Priya, 2016 | India | Women of reproductive age | 60 | RCT | Oral contraceptive<br>Vaginal ring | Weight |

| Study | Country | Population | Sample Size | Study Design | Arms | Outcomes |
|---|---|---|---|---|---|---|
| Quintino-Moro, 2019 | Brazil | Women of reproductive age | 72 | Non-randomized trial | Injectable<br>Non-hormonal IUD | BMI<br>Fat/lean mass<br>Percent fat mass<br>Total mass<br>Weight |
| Rana, 2012 | India | Women of reproductive age | 100 | Prospective cohort | Hormonal IUD<br>Non-hormonal IUD | Ferritin<br>Hematocrit<br>Hemoglobin |
| Ravi, 2021 | India | Women of reproductive age with endometrial hyperplasia with abnormal uterine bleeding (AUB) | 51 | RCT | Hormonal IUD<br>Hormonal IUD + non-contraceptive drug (metformin) | BMI<br>Hemoglobin<br>Weight |
| Rizzo, 2019 | Brazil | Adolescents | 178 | Non-randomized trial | Control (no contraception)<br>Oral contraceptive x 2 | BMI<br>BMI percentile<br>Fat/lean mass<br>Height<br>Total body fat<br>Weight |
| Roy, 2020 | India | Postpartum women | 789 | Non-randomized trial | Non-hormonal IUD<br>Vaginal ring | Hemoglobin |
| Sanam, 2011 | Iran | Women of reproductive age | 100 | RCT | Oral contraceptive x 2 | BMI<br>Weight |
| Sangthawan, 2004 | Thailand | Women of reproductive age | 104 | RCT | Oral contraceptive x 2 | Weight gain |
| Sayed, 2011 | Egypt | Women of reproductive age with heavy menstrual bleeding | 58 | RCT | Hormonal IUD<br>Oral contraceptive | Ferritin<br>Hemoglobin |

| Study | Country | Population | Sample Size | Study Design | Arms | Outcomes |
|---|---|---|---|---|---|---|
| Shabaan, 2011 | Egypt | women self-described heavy menstrual bleeding | 112 | RCT | Hormonal IUD<br>Oral contraceptive | Ferritin<br>Hemoglobin |
| Shahnazi, 2016 | Iran | Women of reproductive age with overweight or obesity | 137 | RCT | Oral contraceptive x 2 | BMI |
| Silva-Filho, 2013 | Brazil | Women of reproductive age with heavy menstrual bleeding | 58 | RCT | Hormonal IUD<br>Thermal balloon ablation | Hemoglobin |
| Soysal,2002 | Turkey | Women over 40 with heavy menstrual bleeding | 72 | RCT | Hormonal IUD<br>Thermal balloon ablation | Hemoglobin |
| Strufaldi, 2010 | Brazil | Women of reproductive age | 101 | RCT | Oral contraceptive x 2 | Abdominal circumference<br>BMI<br>Weight |
| Suthipongse, 2004 | Thailand | Women and adolescents of reproductive age | 120 | RCT | Oral contraceptive x 2 | Weight |
| Suvarna, 2016 | India | Women of reproductive age with PCOS | 46 | Prospective cohort | Non contraceptive drug (metformin)<br>Oral contraceptive | BMI |
| Taha, 2021 | Lebanon | Women of reproductive age with endometriosis | 70 | RCT | Oral contraceptive x 2 | Weight gain |
| Taneepanichskul,2002 | Thailand | Women of reproductive age | 150 | RCT | Oral contraceptive x 2 | Weight |
| Todd, 2020 | South Africa | Women of reproductive age | 205 | RCT | Hormonal IUD + antiretrovirals<br>Non-hormonal IUD | Hemoglobin (mean change) |

| Study | Country | Population | Sample Size | Study Design | Arms | Outcomes |
|---|---|---|---|---|---|---|
| | | | | | Non-hormonal IUD + antiretrovirals | Weight gain<br>Weight loss |
| Vieira, 2012 | Brazil | Women of reproductive age | 50 | RCT | Oral contraceptive<br>Oral contraceptive + non-contraceptive drug (spironolactone) | BMI<br>Waist circumference<br>Weight |
| Wang, 2016 | China | Women of reproductive age with PCOS and a metabolic disorder | 99 | RCT | Oral contraceptive x 2 | BMI<br>Waist-hip ratio |
| Wongwananuruk, 2019 | Thailand | Women of reproductive age | 99 | RCT | Oral contraceptive x 2 | BMI change<br>Waist circumference change<br>Weight change |
| Wu, 2008 | China | Women of reproductive age with obesity and PCOS | 60 | RCT | Oral contraceptive<br>Oral contraceptive + non-contraceptive drug (metformin) | BMI<br>Waist-hip-ratio |
| Yildizhan, 2009 | Turkey | Women of reproductive age | 160 | RCT | Oral contraceptive x 2 | BMI |
| Yildizhan, 2015 | Turkey | Women of reproductive age with PCOS | 120 | RCT | Oral contraceptive x 2 | BMI<br>Waist-hip-ratio |
| Yilmaz, 2005 | Turkey | Pregnant women | 66 | RCT | Abortion drug x 2 | Hemoglobin |
| Zueff, 2016 | Brazil | Women of reproductive age with obesity | 106 | RCT | Hormonal IUD<br>Non-hormonal contraceptive | BMI<br>Waist circumference |

**Supplementary Table 4. Study arms covered by extracted studies**

| Study arm | Number of studies |
|---|---|
| Contraceptive arms | |
| Abortion drug | 7 |
| Abortion surgery | 1 |
| Clinic provision of contraceptives | 1 |
| Hormonal IUD | 26 |
| Hormonal IUD + anti-coagulant therapy | 1 |
| Hormonal IUD + antiretrovirals | 1 |
| Hormonal IUD + non-contraceptive drug | 1 |
| Implant | 12 |
| Implant + antiretroviral therapy | 2 |
| In-home delivery of contraceptives | 1 |
| Injectables | 15 |
| Non-hormonal contraceptive | 8 |
| Non-hormonal IUD | 19 |
| Non-hormonal IUD + antiretrovirals | 1 |
| Non-hormonal IUD + Ulipristal Acetate | 1 |
| Oral contraceptive | 55 |
| Oral contraceptive + non-contraceptive drug | 6 |
| Skin patch | 1 |
| Vaginal ring | 4 |
| Comparators with no contraception | |
| No contraception | 11 |
| Non-contraceptive drug | 4 |
| Supplementation (no contraception) | 3 |
| Thermal balloon ablation | 2 |

**Supplementary Table 5. Outcomes covered by extracted studies**

| Outcomes | Number of studies |
|---|---|
| Abdominal circumference | 7 |
| BMI | 42 |
| BMI change | 1 |
| BMI percentile | 2 |
| BMI percent change | 1 |
| Central obesity | 1 |
| Central peripheral fat ratio | 3 |
| Chance in fat and lean mass | 1 |
| Fat/lean mass ratios | 8 |
| Ferritin | 7 |
| Height | 2 |
| Hematocrit | 5 |
| Hemoglobin | 29 |
| Hemoglobin change | 1 |
| Obesity | 1 |
| Percent android/gynoid fat | 1 |
| Percent fat mass | 1 |
| Total body fat | 4 |
| Total mass | 2 |
| Visceral adiposity | 1 |
| Waist circumference | 10 |
| Waist circumference change | 1 |
| Waist-height ratio | 1 |
| Waist-hip ratio | 13 |
| Waist-hip ratio percent change | 1 |
| Weight | 31 |
| Weight change | 7 |
| Weight gain | 22 |
| Weight loss | 7 |

## Supplementary Table 6. Countries included in extracted studies

| Region | Number of studies |
|---|---|
| **East Asia & Pacific** | **9** |
| China | 4 |
| Malaysia | 1 |
| Thailand | 4 |
| **Europe & Central Asia** | **19** |
| Macedonia | 1 |
| Romania | 1 |
| Russia | 2 |
| Serbia | 1 |
| Turkey | 14 |
| **Latin America & the Caribbean** | **25** |
| Brazil | 23 |
| Cuba | 1 |
| Jamaica | 1 |
| **Middle East & North Africa** | **21** |
| Egypt | 7 |
| Iran | 11 |
| Lebanon | 1 |
| Oman | 1 |
| Tunisia | 1 |
| **North America** | **1** |
| Mexico | 1 |
| **South Asia** | **13** |
| Bangladesh | 2 |
| India | 9 |
| Pakistan | 2 |
| **Sub-Saharan Africa** | **17** |
| Eswatini | 1 |
| Kenya | 2 |
| Malawi | 3 |
| Nigeria | 2 |
| South Africa | 4 |
| Uganda | 3 |
| Zambia | 1 |
| Zimbabwe | 1 |

**Supplementary Table 7. Risk of bias summary in extracted studies**

| Study | Outcome | Risk of Bias Tool | Overall Risk of Bias |
|---|---|---|---|
| Abu Hashim, 2012 | Hemoglobin | ROB2 | Some concerns |
| Abu Hashim, 2012 | Ferritin | ROB2 | Some concerns |
| Afsar, 2005 | Weight | ROBINSE | Very High |
| Afsar, 2005 | BMI | ROBINSE | Very High |
| Afsar, 2005 | Hemoglobin | ROBINSE | Very High |
| Afsar, 2005 | Hematocrit | ROBINSE | Very High |
| Al-Ghashri,2021 | Weight gain | ROBINSE | Very High |
| Amiri, 2020 (a) | BMI | ROB2 | Some concerns |
| Amiri, 2020 (a) | Waist circumference | ROB2 | Some concerns |
| Amiri, 2020 (a) | Waist/hip ratio | ROB2 | Some concerns |
| Amiri, 2020 (a) | Waist/height ratio | ROB2 | Some concerns |
| Amiri, 2020 (a) | Visceral adiposity index | ROB2 | Some concerns |
| Amiri, 2020 (a) | Obesity | ROB2 | Some concerns |
| Amiri, 2020 (a) | Central obesity | ROB2 | Some concerns |
| Amiri, 2020 (b) | Weight | ROB2 | Some concerns |
| Amiri, 2020 (b) | BMI | ROB2 | Some concerns |
| Amiri, 2020 (b) | Waist circumference | ROB2 | Some concerns |
| Amiri, 2020 (b) | Waist/hip ratio | ROB2 | Some concerns |
| Andrade, 2004 | Ferritin | ROBINSI | No information |
| Arowojolu 2003 | Weight loss | ROBINSE | Very High |
| Barham, 2021 | BMI | ROB2-CL | No information |
| Beesham, 2022 (ECHO) | Self reported weight gain | ROB2 | Some concerns |
| Beesham, 2022 (ECHO) | Self reported weight loss | ROB2 | Some concerns |
| Behboudi-Gandevani 2019 | BMI | ROB2 | Low |
| Behboudi-Gandevani 2019 | WHR | ROB2 | Low |
| Behnamfar, 2014 | Self reported weight gain | ROB2 | High |
| Beksinska 2021 | Weight increase/loss (self-reported) | ROB2 | High |
| Beksinska, 2021 | Weight | ROB2 | High |
| Beksinska, 2021 | BMI | ROB2 | High |
| Bilgehan, 2015 | Hemoglobin | ROB2 | Some concerns |

| | | | |
|---|---|---|---|
| Bilgehan, 2015 | Weight | ROB2 | Some concerns |
| Bilgehan, 2015 | BMI | ROB2 | Some concerns |
| Bilgehan, 2015 | Waist/hip ratio | ROB2 | Some concerns |
| Brache, 2021 | Hemoglobin | ROB2 | Low |
| Brache, 2021 | Hematocrit | ROB2 | Low |
| Braga, 2014 | Weight 12 months | ROBINSE | Very High |
| Braga, 2014 | BMI 12 months | ROBINSE | Very High |
| Braga, 2014 | Abdominal circumference 12 months | ROBINSE | Very High |
| Braga, 2014 | Hemoglobin 12 months | ROBINSE | Very High |
| Braga, 2014 | Hematocrit 12 months | ROBINSE | Very High |
| Bright, 2022 | Hemoglobin | ROBINSI | Moderate |
| Brito, 2009 | Weight | ROB2 | Some concerns |
| Brito, 2009 | BMI | ROB2 | Some concerns |
| Brito, 2009 | Waist circumference | ROB2 | Some concerns |
| Brito, 2009 | Hemoglobin | ROB2 | Some concerns |
| Burke, 2018 | Weight change | ROB2 | High |
| Caldeirao, 2022 | Weight | ROBINSI | Critical |
| Caldeirao, 2022 | Height | ROBINSI | Critical |
| Caldeirao, 2022 | BMI | ROBINSI | Critical |
| Caldeirao, 2022 | BMI (percentile) | ROBINSI | Critical |
| Caldeirao, 2022 | Fat mass | ROBINSI | Critical |
| Caldeirao, 2022 | Lean body mass | ROBINSI | Critical |
| Caldeirao, 2022 | Total body fat | ROBINSI | Critical |
| Carbonell, 2008 | Hemoglobin | ROB2 | Some concerns |
| Chappell, 2017 | Weight gain (self-reported) | ROBINSI | Critical |
| Chappell, 2019 | Weight gain | ROBINSE | Very High |
| Cursino, 2018 | Weight | ROBINSI | Serious |
| Cursino, 2018 | BMI | ROBINSI | Serious |
| Cursino, 2018 | Fat mass | ROBINSI | Serious |
| Cursino, 2018 | Total body fat | ROBINSI | Serious |
| Cursino, 2018 | Lean body mass | ROBINSI | Serious |
| Cursino, 2018 | Waist/hip ratio | ROBINSI | Serious |
| Dabash, 2010 | Hemoglobin change | ROB2 | Low |

| | | | |
|---|---|---|---|
| Dal'Ava, 2012 | Weight | ROBINSI | Moderate |
| Dal'Ava, 2012 | central-peripheral fat ratio | ROBINSI | Moderate |
| Dal'Ava, 2012 | change in Fat mass | ROBINSI | Moderate |
| Dal'Ava, 2012 | change in Lean mass | ROBINSI | Moderate |
| Dal'Ava, 2014 | Weight | ROBINSI | Moderate |
| Dal'Ava, 2014 | Fat mass | ROBINSI | Moderate |
| Dal'Ava, 2014 | Lean mass | ROBINSI | Moderate |
| Dal'Ava, 2014 | Central-peripheral fat ratio | ROBINSI | Moderate |
| de Morais, 2014 | Weight | ROBINSI | Critical |
| de Morais, 2014 | BMI | ROBINSI | Critical |
| de Morais, 2014 | Abdominal circumference | ROBINSI | Critical |
| de Morais, 2014 | Hemoglobin | ROBINSI | Critical |
| Deo, 2021 | BMI | ROB2 | Some concerns |
| Deo, 2021 | Waist circumference | ROB2 | Some concerns |
| Deo, 2021 | Waist/hip ratio | ROB2 | Some concerns |
| Dos Santos Quaresma, 2022 | Weight | ROBINSI | Serious |
| Dos Santos Quaresma, 2022 | Waist circumference | ROBINSI | Serious |
| Dos Santos Quaresma, 2022 | Abdominal circumference | ROBINSI | Serious |
| Dos Santos Quaresma, 2022 | Fat mass | ROBINSI | Serious |
| Dos Santos Quaresma, 2022 | Lean body mass | ROBINSI | Serious |
| Erol, 2014 | Hemoglobin | ROBINSI | Critical |
| Fekih 2010 | Hemoglobin | ROB2 | Some concerns |
| Fekih 2010 | Hematocrit | ROB2 | Some concerns |
| Franceschini 2012 | Weight | ROBINSI | Critical |
| Franceschini 2012 | BMI | ROBINSI | Critical |
| Gallo, 2016 | Weight change | ROB2 | Some concerns |
| Gallo, 2016 | perceived Weight gain | ROB2 | High |
| Gallo, 2016 | perceived Weight loss | ROB2 | High |
| Giribela, 2012 | Weight | ROBINSE | Very High |
| Giribela, 2012 | BMI | ROBINSE | Very High |
| Gomes, 2012 | BMI | ROBINSI | Critical |
| Gomes, 2012 | Waist circumference | ROBINSI | Critical |
| Guang-Sheng, 2010 | Weight gain | ROB2 | High |

| | | | |
|---|---|---|---|
| Guang-Sheng, 2010 | Weight change | ROB2 | High |
| Gurmu, 2022 | Hemoglobin | ROBINSE | Very High |
| Haddad, 2013 | Weight gain | ROB2 | High |
| Haddad, 2013 | Weight loss | ROB2 | High |
| Hassanin, 2021 | Weight gain | ROB2 | Low |
| Hernandez-Juarez, 2014 | Weight | ROBINSI | Critical |
| Hernandez-Juarez, 2014 | BMI | ROBINSI | Critical |
| Hernandez-Juarez, 2014 | Abdominal circumference | ROBINSI | Critical |
| Hou, 2010 | Hemoglobin | ROB2 | Some concerns |
| Hubacher, 2015 | Weight gain | ROBINSI | Critical |
| Hubacher, 2015 | Weight loss | ROBINSI | Critical |
| Ibrahim, 2019 | Hemoglobin | ROB2 | Some concerns |
| Ibrahim, 2019 | Weight change | ROBINSE | Low |
| Ilyin 2021 | Hemoglobin | ROB2 | Low |
| Ilyin 2021 | Ferritin | ROB2 | Low |
| Ilyin 2021 | BMI | ROB2 | Low |
| Ilyin 2021 | Body Weight | ROB2 | Low |
| Ilyin 2021 | Weight increased (self-reported) | ROB2 | High |
| Iwata, 2015 | BMI | ROBINSI | Critical |
| Jamali, 2014 | Weight gain (self-reported) | ROBINSI | Critical |
| Jimoh 2021 | BMI | ROBINSI | Critical |
| Kachhawa, 2021 | Weight | ROB2 | Low |
| Kachhawa, 2021 | BMI | ROB2 | Low |
| Kachhawa, 2021 | Waist/hip ratio | ROB2 | Low |
| Kahraman, 2014 | BMI change | ROB2 | High |
| Kahraman, 2014 | WHR change | ROB2 | High |
| Kakaire, 2015 | Weight change | ROB2 | Low |
| Karimi-Zarchi,2013 | Weight change | ROB2 | High |
| Kashanian, 2010 | Weight increase (continuous) | ROB2 | Some concerns |
| Kashanian, 2010 | Weight decrease | ROB2 | Some concerns |
| Kashanian, 2010 | Weight increase (continuous) | ROB2 | Some concerns |
| Kavasoglu, 2020 | Hemoglobin | ROB2 | Some concerns |
| Kavasoglu, 2020 | Weight gain | ROB2 | High |

| | | | |
|---|---|---|---|
| Kelekci, 2012a | Hemoglobin | ROBINSI | Critical |
| Kelekci, 2012b | Weight change | ROB2 | High |
| Kilic, 2011 | BMI | ROB2 | Some concerns |
| Kilic, 2011 | Abdominal circumference | ROB2 | Some concerns |
| Kumar, 2005 | Hemoglobin | ROBINSI | Serious |
| Kumar, 2018 | Weight | ROB2 | Some concerns |
| Kumar, 2018 | BMI | ROB2 | Some concerns |
| Kumar, 2018 | Total body fat | ROB2 | Some concerns |
| Kumar, 2018 | Percent android/gynoid fat | ROB2 | Some concerns |
| Machado, 2006 | total body Weight | ROB2 | High |
| Machado, 2006 | fat free mass | ROB2 | High |
| Machado, 2006 | Fat mass | ROB2 | High |
| Malik, 2020 | Hemoglobin | ROB2 | Some concerns |
| Mawet, 2014 | Hemoglobin | ROB2 | High |
| Mawet, 2014 | Ferritin | ROB2 | High |
| Mehrabian 2016 | Waist circumference | ROB2 | Low |
| Mehrabian 2016 | BMI | ROB2 | Low |
| Melhado-Kimura, 2018 | Weight | ROBINSI | Critical |
| Melhado-Kimura, 2018 | BMI | ROBINSI | Critical |
| Melhado-Kimura, 2018 | Total mass | ROBINSI | Critical |
| Melhado-Kimura, 2018 | Fat mass | ROBINSI | Critical |
| Melhado-Kimura, 2018 | Fat mass | ROBINSI | Critical |
| Melhado-Kimura, 2018 | Lean mass | ROBINSI | Critical |
| Melhado-Kimura, 2018 | Central-peripheral fat ratio | ROBINSI | Critical |
| Mohamed, 2011 | Weight gain | ROB2 | High |
| Momeni, 2020 | BMI | ROBINSE | Very High |
| Momeni, 2020 | WHR | ROBINSE | Very High |
| Moreira, 2020 | Hemoglobin | ROBINSI | Critical |
| Nakalema, 2022 | Weight gain | ROBINSI | Serious |
| Nisenbaum, 2014 | BMI | ROBINSI | Critical |
| Nooh, 2016 | Weight gain | ROB2 | High |
| Oderich, 2012 | BMI | ROBINSI | Critical |
| Onwuka 2020 | Hemoglobin/anemia | ROBINSE | Very High |

| | | | |
|---|---|---|---|
| Onwuka 2020 | GWG | ROBINSE | Very High |
| Ortayli, 2001 | Weight | ROBINSI | Moderate |
| Ozay, 2016 | BMI | ROBINSI | Critical |
| Ozay, 2016 | BMI | ROBINSI | Critical |
| Ozdemir, 2008 | BMI | ROB2 | Some concerns |
| Ozdemir, 2008 | Waist-hip ratio | ROB2 | Some concerns |
| Ozdemir, 2008 | Fasting glucose | ROB2 | Some concerns |
| Priya, 2016 | Weight | ROB2 | High |
| Quintino-Moro, 2019 | Weight | ROBINSI | Critical |
| Quintino-Moro, 2019 | total mass | ROBINSI | Critical |
| Quintino-Moro, 2019 | Fat mass | ROBINSI | Critical |
| Quintino-Moro, 2019 | % Fat mass | ROBINSI | Critical |
| Quintino-Moro, 2019 | Lean mass | ROBINSI | Critical |
| Rana, 2012 | Hemoglobin | ROBINSI | Moderate |
| Rana, 2012 | Ferritin | ROBINSI | Moderate |
| Rana, 2012 | Hematocrit | ROBINSI | Moderate |
| Ravi, 2021 | Hemoglobin | ROB2 | Low |
| Ravi, 2021 | BMI | ROB2 | Low |
| Ravi, 2021 | Weight | ROB2 | Low |
| Rizzo, 2019 | Weight | ROBINSI | Serious |
| Rizzo, 2019 | Height | ROBINSI | Serious |
| Rizzo, 2019 | BMI | ROBINSI | Serious |
| Rizzo, 2019 | BMI (percentile) | ROBINSI | Serious |
| Rizzo, 2019 | Fat mass | ROBINSI | Serious |
| Rizzo, 2019 | Lean body mass | ROBINSI | Serious |
| Rizzo, 2019 | Total body fat | ROBINSI | Serious |
| Roy, 2020 | Hemoglobin | ROBINSI | Critical |
| Sanam, 2011 | Weight | ROB2 | Some concerns |
| Sanam, 2011 | BMI | ROB2 | Some concerns |
| Sanga, 2020 | Hemoglobin (Anemia) | ROBINSE | Very High |
| Sangthawan, 2004 | Weight change | ROB2 | High |
| Sayed, 2011 | Hemoglobin | ROB2 | High |
| Sayed, 2011 | Ferritin | ROB2 | High |

| | | | |
|---|---|---|---|
| Shabaan, 2011 | Hemoglobin | ROB2 | Some concerns |
| Shabaan, 2011 | Ferritin | ROB2 | Some concerns |
| Shahnazi, 2016 | BMI | ROB2 | Low |
| Silva-Filho, 2013 | Hemoglobin | ROB2 | High |
| Soysol, 2002 | Hemoglobin | ROB2 | Some concerns |
| Strufaldi, 2010 | Weight | ROB2 | Some concerns |
| Strufaldi, 2010 | BMI | ROB2 | Some concerns |
| Strufaldi, 2010 | Abdominal circumference | ROB2 | Some concerns |
| Suthipongse, 2004 | Weight | ROB2 | High |
| Suvarna 2016 | BMI | ROBINSI | Critical |
| Taha, 2021 | Weight gain | ROB2 | High |
| Taneepanichskul, 2002 | Weight | ROB2 | Some concerns |
| Todd, 2020 | Hemoglobin | ROB2 | Low |
| Todd, 2020 | Weight gain | ROB2 | High |
| Todd, 2020 | Weight loss | ROB2 | High |
| Vieira, 2012 | Weight | ROB2 | Some concerns |
| Vieira, 2012 | BMI | ROB2 | Some concerns |
| Vieira, 2012 | Waist circumference | ROB2 | Some concerns |
| Wang, 2016 | BMI | ROB2 | High |
| Wang, 2016 | WHR | ROB2 | High |
| Wongwananuruk, 2019 | Weight change | ROB2 | Low |
| Wongwananuruk, 2019 | BMI change | ROB2 | Low |
| Wongwananuruk, 2019 | Waist circumference change | ROB2 | Low |
| Wu, 2008 | BMI | ROB2 | Some concerns |
| Wu, 2008 | Waist-hip ratio | ROB2 | Some concerns |
| Yildizhan, 2009 | BMI | ROB2 | High |
| Yildizhan, 2015 | BMI | ROB2 | Some concerns |
| Yildizhan, 2015 | Waist/hip ratio | ROB2 | Some concerns |
| Yilmaz 2005 | Hemoglobin decrease | ROB2 | Low |
| Zueff, 2016 | BMI | ROB2 | Some concerns |
| Zueff, 2016 | Waist circumference | ROB2 | Some concerns |

## Supplementary Table 8: GRADE evidence profile for comparison of hormonal intrauterine device to oral contraceptives for outcome of hemoglobin

**Question:** Hormonal Intrauterine Device (IUD) compared to Oral Contraceptives for Nutrition-Related Outcomes in Women and Adolescent Girls of Reproductive Age

**Setting:** Low- and Middle-Income Countries

**Bibliography:**


Kavasoglu, A., & Gocmen, A. (2020). Progesterone treatment preference in heavy menstrual bleeding: Per Os or Levonorgestrel releasing intrauterine device. Kuwait Medical Journal.

Malik, F., Sara, S., & Kasi, R. (2020). Comparative Trial of Levonorgestrel Intrauterine System and Norethisterone for Treatment of Idiopathic Menorrhagia (Vol. 14, Issue 4).

Sayed, G. H., Zakherah, M. S., El-Nashar, S. A., & Shaaban, M. M. (2011). A randomized clinical trial of a levonorgestrel-releasing intrauterine system and a low-dose combined oral contraceptive for fibroid-related menorrhagia. International Journal of Gynecology and Obstetrics, 112(2), 126–130. https://doi.org/10.1016/j.ijgo.2010.08.009

Shabaan, M. M., Zakherah, M. S., El-Nashar, S. A., & Sayed, G. H. (2011). Levonorgestrel-releasing intrauterine system compared to low dose combined oral contraceptive pills for idiopathic menorrhagia: A randomized clinical trial. Contraception, 83(1), 48–54. https://doi.org/10.1016/j.contraception.2010.06.011


| Certainty assessment | | | | | | | № of patients | | Effect | | Certainty | Importance |
|---|---|---|---|---|---|---|---|---|---|---|---|---|
| № of studies | Study design | Risk of bias | Inconsistency | Indirectness | Imprecision | Other considerations | Hormonal Intrauterine Device (IUD) | Oral Contraceptives | Relative (95% CI) | Absolute (95% CI) | | |
| **Hemoglobin (assessed with: g/dL)** | | | | | | | | | | | | |
| 4 | randomised trials | serious[a] | serious[b] | serious[c] | serious[d] | strong association | 186 | 152 | - | MD **1.25 g/dL higher** (0.38 higher to 2.12 higher) | ⨁◯◯◯ Very low | CRITICAL |

**CI:** confidence interval; **MD:** mean difference

## *Explanations*

a. All studies had some concerns for the risk of bias assessment except one, which was judged as high risk, as per the RoB2 tool.

b. Heterogeneity was found to be at 92%.

c. The population for three studies out of four is women with heavy menstrual bleeding.

d. Less than 400 participants.

## Supplementary Table 9: GRADE evidence profile for comparison of any hormonal contraceptive to any non-contraceptive for outcome of BMI

**Question:** Any Hormonal Contraceptive compared to Any Non-Contraceptive for Nutrition-Related Outcomes in Women and Adolescent Girls of Reproductive Age

**Setting:** Low- and Middle-Income Countries

**Bibliography:**

Behboudi-Gandevani, S., Abtahi, H., Saadat, N., Tohidi, M., & Ramezani Tehrani, F. (2019). Effect of phlebotomy versus oral contraceptives containing cyproterone acetate on the clinical and biochemical parameters in women with polycystic ovary syndrome: A randomized controlled trial. Journal of Ovarian Research, 12(1). https://doi.org/10.1186/s13048-019-0554-9

Deo, S., Pandey, K., Kumari, P., Deo, N., Jaiswar, S. P., Ahmad, M. K., & Agarwal, M. (2021). Effectiveness of Myo-inositol and Combined Oral Contraceptives in Adolescent and Young Women with PCOS. SSR Institute of International Journal of Life Sciences, 7(2), 2763–2773. https://doi.org/10.21276/ssr-iijls.2021.7.2.1

Kachhawa, G., Senthil Kumar, K. V., Kulshrestha, V., Khadgawat, R., Mahey, R., & Bhatla, N. (2022). Efficacy of myo-inositol and d-chiro-inositol combination on menstrual cycle regulation and improving insulin resistance in young women with polycystic ovary syndrome: A randomized open-label study. International Journal of Gynecology and Obstetrics, 158(2), 278–284. https://doi.org/10.1002/ijgo.13971

Kilic, S., Yilmaz, N., Zulfikaroglu, E., Erdogan, G., Aydin, M., & Batioglu, S. (2011). Inflammatory-metabolic parameters in obese and nonobese normoandrogenemic polycystic ovary syndrome during metformin and oral contraceptive treatment. Gynecological Endocrinology, 27(9), 622–629. https://doi.org/10.3109/09513590.2010.530706

Kumar, Y., Kotwal, N., Singh, Y., Upreti, V., Somani, S., & Hari Kumar, KV. S. (2018). A randomized, controlled trial comparing the metformin, oral contraceptive pills and their combination in patients with polycystic ovarian syndrome. Journal of Family Medicine and Primary Care, 7(3), 551. https://doi.org/10.4103/jfmpc.jfmpc_83_17

Mehrabian, F., Ghasemi-Tehrani, H., Mohamadkhani, M., Moeinoddini, M., & Karimzadeh, P. (2016). Comparison of the effects of metformin, flutamide plus oral contraceptives, and simvastatin on the metabolic consequences of polycystic ovary syndrome. Journal of Research in Medical Sciences, 21(1). https://doi.org/10.4103/1735-1995.177354

Ozay, A. C., Emekci Ozay, O., Okyay, R. E., Cagliyan, E., Kume, T., & Gulekli, B. (2016). Different Effects of Myoinositol plus Folic Acid versus Combined Oral Treatment on Androgen Levels in PCOS Women. International Journal of Endocrinology, 2016. https://doi.org/10.1155/2016/3206872

| Certainty assessment | | | | | | | № of patients | | Effect | | Certainty | Importance |
|---|---|---|---|---|---|---|---|---|---|---|---|---|
| № of studies | Study design | Risk of bias | Inconsistency | Indirectness | Imprecision | Other considerations | Any Hormonal Contraceptive | Any Non-Contraceptive | Relative (95% CI) | Absolute (95% CI) | | |
| **Body Mass Index (assessed with: weight/(height²))** | | | | | | | | | | | | |
| 7 | randomised trials | not serious | serious[a] | serious[b] | not serious | none | 250 | 244 | - | MD **0.28 kg/m² lower** (0.52 lower to 0.04 lower) | ⨁⨁◯◯ Low | IMPORTANT |

**CI:** confidence interval; **MD:** mean difference

## *Explanations*

a. Test of heterogeneity was moderate with $I^2$: 44.67%.

b. Population for five out of seven studies was women with PCOS.

## Supplementary Table 10: GRADE evidence profile for comparison of any hormonal contraceptive vs any non-hormonal contraceptive for outcome of BMI

**Question:** Any Hormonal Contraceptive compared to Any Non-Hormonal Contraceptive in Nutrition-Related Outcomes for Women and Adolescent Girls of Reproductive Age

**Setting:** Low- and Middle-Income Countries

**Bibliography:**


Beesham, I., Bosman, S., Beksinska, M., Scoville, C. W., Smit, J., & Nanda, K. (2022). Contraceptive method preference and reasons for contraceptive discontinuation among women randomized to intramuscular depot medroxyprogesterone acetate, a copper intrauterine device or a levonorgestrel implant: Findings from Durban, South Africa. Contraception, 108, 37–43. https://doi.org/10.1016/j.contraception.2021.11.002

Beksinska, M., Issema, R., Beesham, I., Lalbahadur, T., Thomas, K., Morrison, C., Hofmeyr, G. J., Steyn, P. S., Mugo, N., Palanee-Phillips, T., Ahmed, K., Nair, G., Baeten, J. M., & Smit, J. (2021). Weight change among women using intramuscular depot medroxyprogesterone acetate, a copper intrauterine device, or a levonorgestrel implant for contraception: Findings from a randomised, multicentre, open-label trial. EClinicalMedicine, 34. https://doi.org/10.1016/j.eclinm.2021.100800

Bilgehan, F., Dilbaz, B., Karadag, B., & Deveci, C. D. (2015). Comparison of copper intrauterine device with levonorgestrel-bearing intrauterine system for post-abortion contraception. Journal of Obstetrics and Gynaecology Research, 41(9), 1426–1432. https://doi.org/10.1111/jog.12747

Cursino, K., de Lima, G. A., de Nazaré Silva dos Santos, P., Pavin, E. J., Bahamondes, L., & Fernandes, A. (2018). Subclinical cardiovascular disease parameters after one year in new users of depot medroxyprogesterone acetate compared to copper-IUD. European Journal of Contraception and Reproductive Health Care, 23(3), 201–206. https://doi.org/10.1080/13625187.2018.1455087

Franceschini, S. A., Vieira, C. S., Martins, W. P., França, J. B., & Ferriani, R. A. (2013). Effects of combined oral contraceptives containing levonorgestrel or chlormadinone on the endothelium. Contraception, 87(6), 766–772. https://doi.org/10.1016/j.contraception.2012.09.023

Giribela, C. R. G., Melo, N. R., Silva, R. C. G., Hong, V. M., Guerra, G. M., Baracat, E. C., & Consolim-Colombo, F. M. (2012). A combined oral contraceptive containing drospirenone changes neither endothelial function nor hemodynamic parameters in healthy young women: A prospective clinical trial. Contraception, 86(1), 35–41. https://doi.org/10.1016/j.contraception.2011.08.017

Haddad, L. B., Cwiak, C., Jamieson, D. J., Feldacker, C., Tweya, H., Hosseinipour, M., Hoffman, I., Bryant, A. G., Stuart, G. S., Noah, I., Mulundila, L., Samala, B., Mayne, P., & Phiri, S. (2013). Contraceptive adherence among HIV-infected women in Malawi: A randomized controlled trial of the copper intrauterine device and depot medroxyprogesterone acetate. Contraception, 88(6), 737–743. https://doi.org/10.1016/j.contraception.2013.08.006

Morais, T. L. De, Giribela, C., Nisenbaum, M. G., Guerra, G., Mello, N., Baracat, E., & Consolim-Colombo, F. M. (2014). Effects of a contraceptive containing drospirenone and ethinylestradiol on blood pressure, metabolic profile and neurohumoral axis in hypertensive women at reproductive age. European Journal of Obstetrics and Gynecology and Reproductive Biology, 182, 113–117. https://doi.org/10.1016/j.ejogrb.2014.09.006

Nisenbaum, M. G., De Melo, N. R., Giribela, C. R. G., De Morais, T. L., Guerra, G. M., De Angelis, K., Mostarda, C., Baracat, E. C., & Consolim-Colombo, F. M. (2014). Effects of a contraceptive containing drospirenone and ethinyl estradiol on blood pressure and autonomic tone: A prospective controlled clinical trial. European Journal of Obstetrics and Gynecology and Reproductive Biology, 175(1), 62–66. https://doi.org/10.1016/j.ejogrb.2014.01.006

Oderich, C. L., Wender, M. C. O., Lubianca, J. N., Santos, L. M., & De Mello, G. C. (2012). Impact of etonogestrel-releasing implant and copper intrauterine device on carbohydrate metabolism: A comparative study. Contraception, 85(2), 173–176. https://doi.org/10.1016/j.contraception.2011.05.018

Quintino-Moro, A., Zantut-Wittmann, D. E., Silva dos Santos, P. de N., Melhado-Kimura, V., da Silva, C. A., Bahamondes, L., & Fernandes, A. (2019). Thyroid function during the first year of use of the injectable contraceptive depot medroxyprogesterone acetate. European Journal of Contraception and Reproductive Health Care, 24(2), 102–108. https://doi.org/10.1080/13625187.2018.1559284

Todd, C. S., Jones, H. E., Langwenya, N., Hoover, D. R., Chen, P. L., Petro, G., & Myer, L. (2020). Safety and continued use of the levonorgestrel intrauterine system as compared with the copper intrauterine device among women living with HIV in South Africa: A randomized controlled trial. PLoS Medicine, 17(5). https://doi.org/10.1371/journal.pmed.1003110

Zueff, L. F. N., Melo, A. S. de, Vieira, C. S., Martins, W. P., & Ferriani, R. A. (2017). Cardiovascular risk markers among obese women using the levonorgestrel-releasing intrauterine system: A randomised controlled trial. Obesity Research and Clinical Practice, 11(6), 687–693. https://doi.org/10.1016/j.orcp.2017.06.001

| Certainty assessment | | | | | | | № of patients | | Effect | | Certainty | Importance |
|---|---|---|---|---|---|---|---|---|---|---|---|---|
| № of studies | Study design | Risk of bias | Inconsistency | Indirectness | Imprecision | Other considerations | Any Hormonal Contraceptive | Any Non-Hormonal Contraceptive | Relative (95% CI) | Absolute (95% CI) | | |
| **Body Mass Index (assessed with: weight/heigh^2)** | | | | | | | | | | | | |
| 3 Zueff 2016, Bilgehan 2015, Beksinska 2021 | randomised trials | serious[a] | serious[b] | serious[c] | not serious | none | 2455 | 2426 | - | MD **0.17 kg/m^2 lower** (0.53 lower to 0.2 higher) | ⨁◯◯◯ Very low | IMPORTANT |
| **Body Mass Index (assessed with: weight/heigh^2)** | | | | | | | | | | | | |
| 3 Zueff 2016, Bilgehan 2015, Beksinska 2021 | randomised trials | serious[a] | very serious[d] | serious[c] | not serious | none | 2376 | 2426 | - | MD **0.16 kg/m^2 lower** (0.53 lower to 0.21 higher) | ⨁◯◯◯ Very low | IMPORTANT |
| **Body Mass Index (assessed with: weight/height^2)** | | | | | | | | | | | | |
| 7 Cursino 2018, Oderich 2012, Nisenbaum, 2014, Quintino-Moro 2019, Morais 2014, Franceschini 2013, Giribela 2012 | observational studies | extremely serious[e] | not serious | not serious | not serious | all plausible residual confounding would suggest spurious effect, while no effect was observed | 193 | 167 | - | MD **0.05 kg/m^2 higher** (0.16 lower to 0.25 higher) | ⨁⨁◯◯ Low | IMPORTANT |
| **Body Mass Index (assessed with: weight/height^2)** | | | | | | | | | | | | |
| 7 Cursino 2018, Oderich 2012, Nisenbaum, 2014, Quintino-Moro 2019, Morais 2014, Franceschini 2013, Giribela 2012 | observational studies | extremely serious[e] | not serious | not serious | not serious | all plausible residual confounding would suggest spurious effect, while no effect was observed | 193 | 167 | - | MD **0.08 kg/m^2 higher** (0.13 lower to 0.28 higher) | ⨁⨁◯◯ Low | IMPORTANT |
| **Weight Gain (assessed with: Self-reported by participants)** | | | | | | | | | | | | |
| 3 Haddad 2013, Beesham 2022, Todd 2020 | randomised trials | serious[f] | not serious | serious[g] | not serious | none | 74/255 (29.0%) | 69/250 (27.6%) | **OR 1.09** (0.69 to 1.73) | **18 more per 1,000** (from 68 fewer to 121 more) | ⨁⨁◯◯ Low | IMPORTANT |
| **Weight Gain (assessed with: Self-reported by participants)** | | | | | | | | | | | | |

| Certainty assessment | | | | | | | № of patients | | Effect | | Certainty | Importance |
|---|---|---|---|---|---|---|---|---|---|---|---|---|
| № of studies | Study design | Risk of bias | Inconsistency | Indirectness | Imprecision | Other considerations | Any Hormonal Contraceptive | Any Non-Hormonal Contraceptive | Relative (95% CI) | Absolute (95% CI) | | |
| 3 Haddad 2013, Beesham 2022, Todd 2020 | randomised trials | serious[f] | not serious | serious[g] | not serious | none | 77/256 (30.1%) | 69/250 (27.6%) | **OR 1.13** (0.72 to 1.79) | **25 more per 1,000** (from 61 fewer to 130 more) | ⨁⨁◯◯ Low | IMPORTANT |
| **Weight Loss (assessed with: Self-reported by participants)** | | | | | | | | | | | | |
| 3 Haddad 2013, Beesham 2022, Todd 2020 | randomised trials | serious[f] | not serious | serious[h] | not serious | none | 41/255 (16.1%) | 51/250 (20.4%) | **OR 0.63** (0.37 to 1.09) | **65 fewer per 1,000** (from 117 fewer to 14 more) | ⨁⨁◯◯ Low | IMPORTANT |
| **Weight Loss (assessed with: Self-reported by participants)** | | | | | | | | | | | | |
| 3 Haddad 2013, Beesham 2022, Todd 2020 | randomised trials | serious[f] | not serious | serious[h] | not serious | none | 38/256 (14.8%) | 51/250 (20.4%) | **OR 0.59** (0.34 to 1.02) | **73 fewer per 1,000** (from 124 fewer to 3 more) | ⨁⨁◯◯ Low | IMPORTANT |

**CI:** confidence interval; **MD:** mean difference; **OR:** odds ratio

## Explanations

a. All risk of bias assessments were completed using RoB2 tool. One study had high levels of concerns, and two studies had some concerns. The study with high levels of concerns was the study with the most participants and larger weight.

b. There was medium heterogeneity between the studies, with I^2=74.23%.

c. Populations were vastly different, including women who underwent voluntary termination of pregnancy, obese women, and healthy women.

d. There was high heterogeneity between the studies with I^2: 75.35%

e. One study was found to have serious risk of bias, while all others had critical risk of bias, as per the ROBINS-I tool for analysis of risk of bias in non-randomized interventional studies.

f. The two studies with the larger weight were found to be at high risk of bias, while the third study was found to have some concerns, as assessed by the RoB2 tool.

g. Women in study with larger weight were HIV-positive. Weight gain is self-reported and highly subjected to bias.

h. Women in study with larger weight were HIV-positive. Weight loss is self-reported and highly subjected to bias.

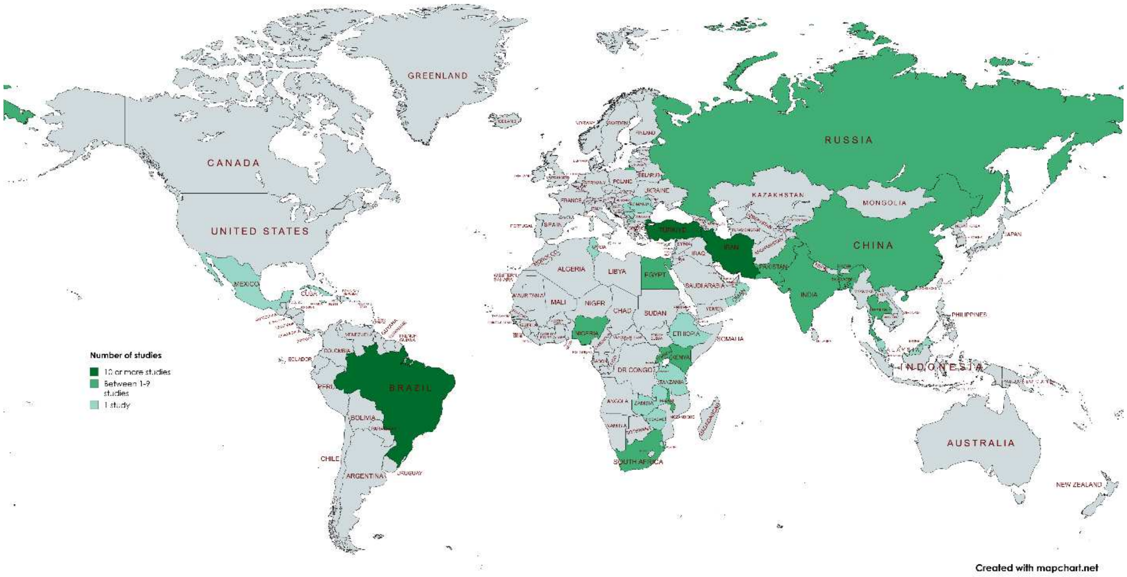


**Supplementary Figure 1.** Map of study areas

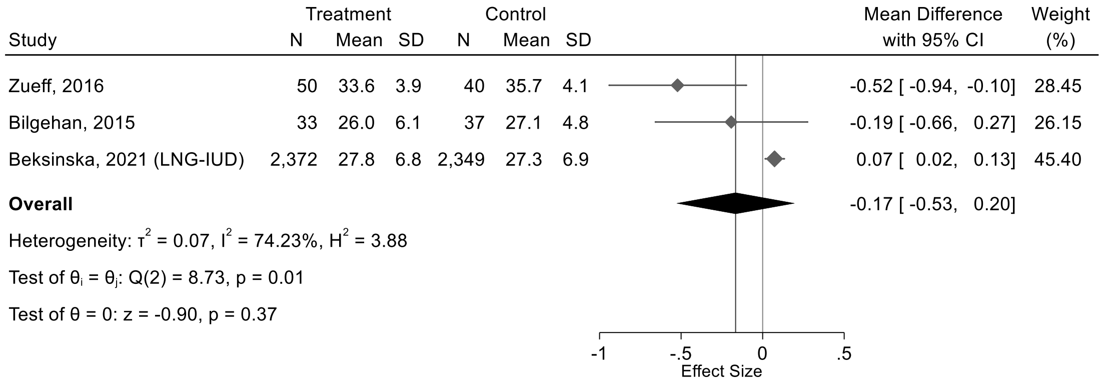


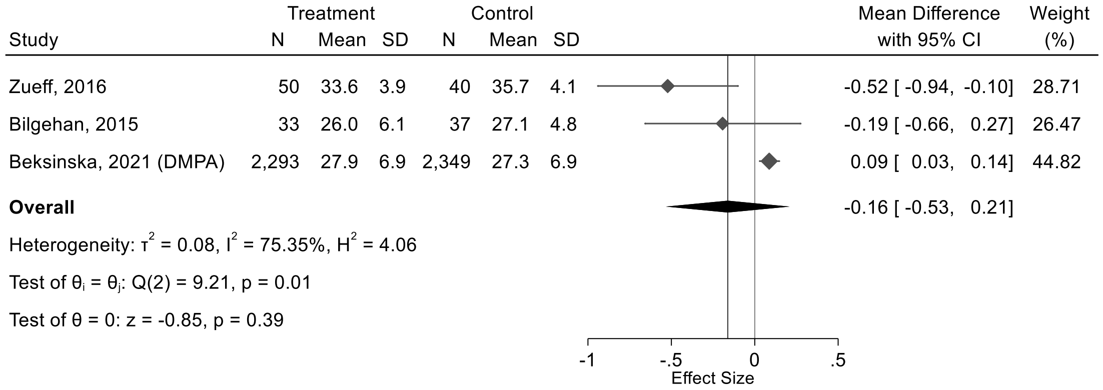


**Supplementary Figure 2.** A) Forest plot presenting the random-effects meta-analysis of RCTs reporting the impact of any hormonal contraceptive method (treatment) compared to non-hormonal contraceptive methods (controls) on BMI outcomes (kg/m$^2$) including only the LNG-IUD arm from Beksinska, 2021. B) Forest plot presenting the random-effects meta-analysis of RCTs reporting the impact of any hormonal contraceptive method (treatment) compared to non-hormonal contraceptive methods (controls) on BMI outcomes (kg/m$^2$) including only the DMPA arm from Beksinska, 2021.

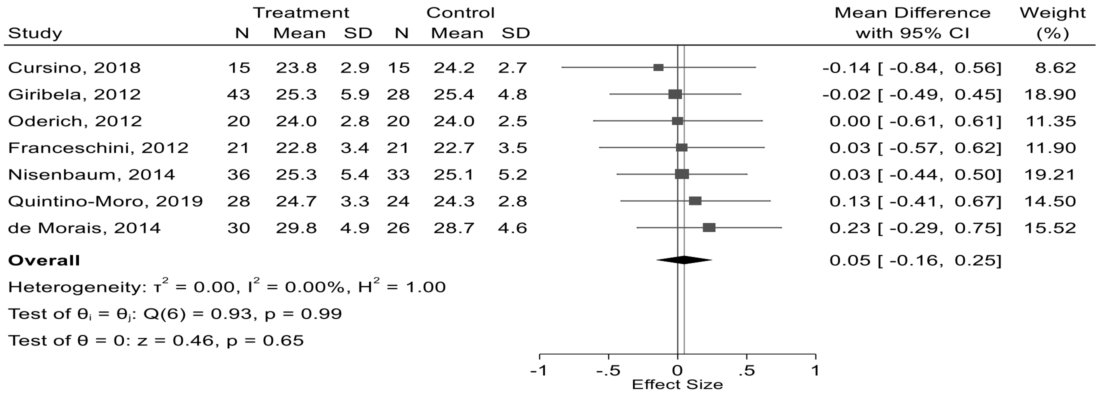


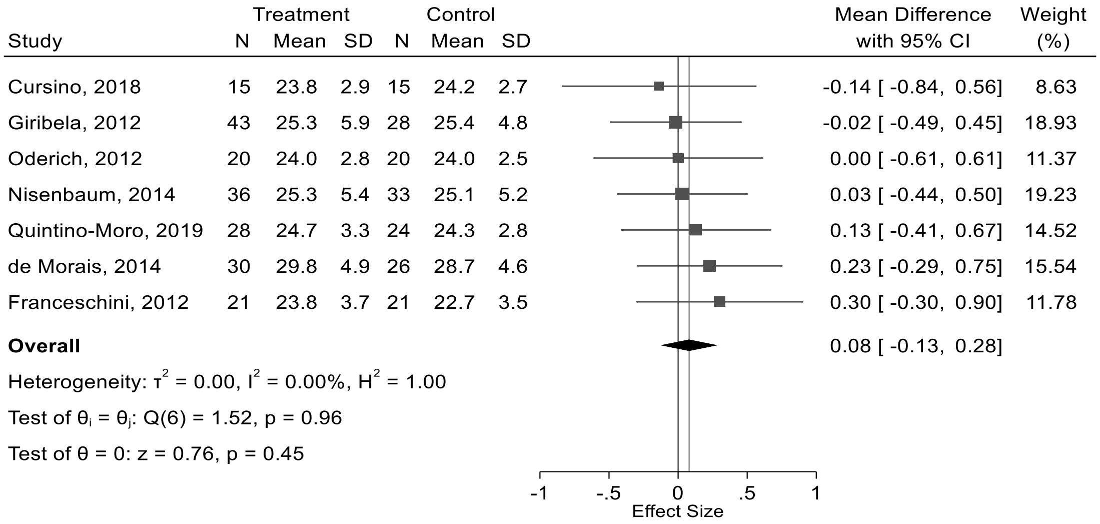


**Supplementary Figure 3.A)** Forest plot presenting the random-effects meta-analysis of non-randomized studies reporting the impact of any hormonal contraceptive method (treatment) compared to non-hormonal contraceptive methods (controls) on BMI outcomes (kg/m²) using the ethinylestradiol/levonorgestrel arm from Franceschini, 2012. **B)** Forest plot presenting the random-effects meta-analysis of non-randomized studies reporting the impact of any hormonal contraceptive method (treatment) compared to non-hormonal contraceptive methods (controls) on BMI outcomes (kg/m²) using the ethinylestradiol/ chlormadinone acetate arm from Franceschini, 2012.

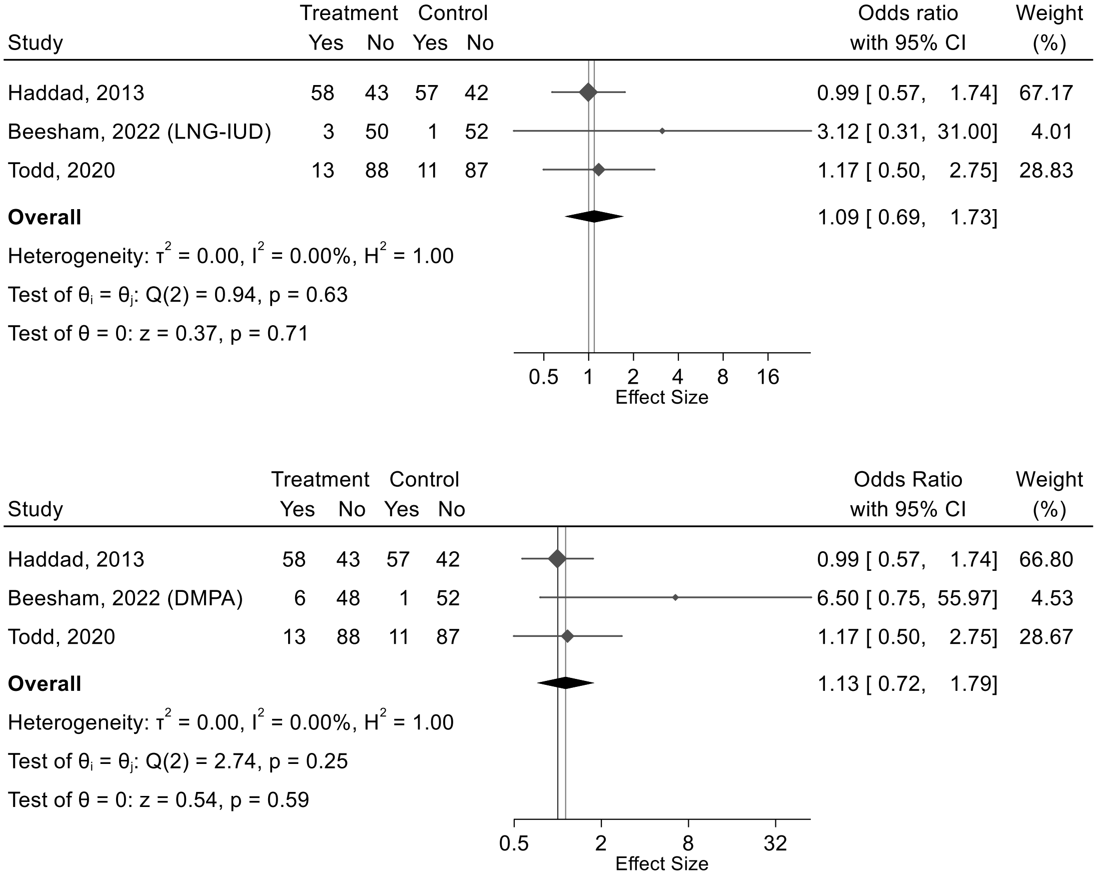


**Supplementary Figure 4** A) Forest plot presenting the random-effects meta-analysis odds ratios of randomized controlled trials reporting the impact of hormonal contraceptives (treatment) compared to non-hormonal contraceptives (control) on self-reported weight gain using only the LNG-IUD arm from Beesham 2022. B) Forest plot presenting the random-effects meta-analysis odds ratios of randomized controlled trials reporting the impact of hormonal contraceptives (treatment) compared to non-hormonal contraceptives (control) on self-reported weight gain using only the DMPA arm from Beesham 2022.

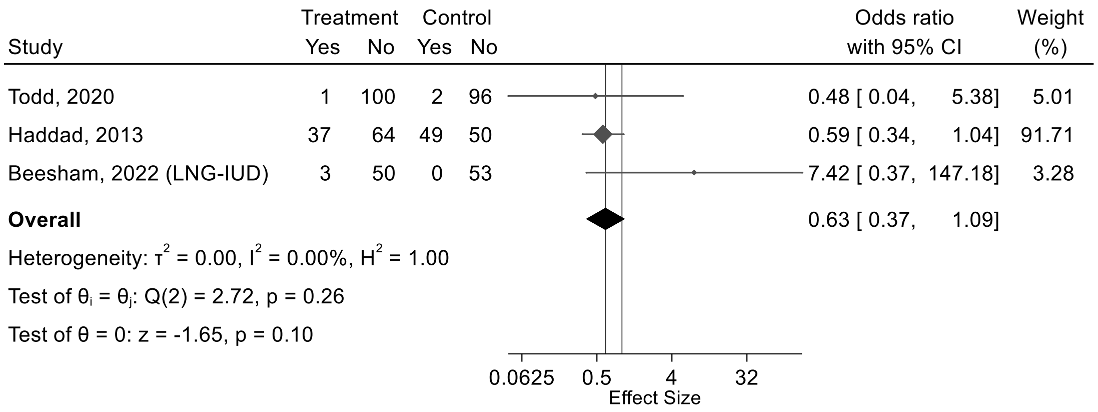


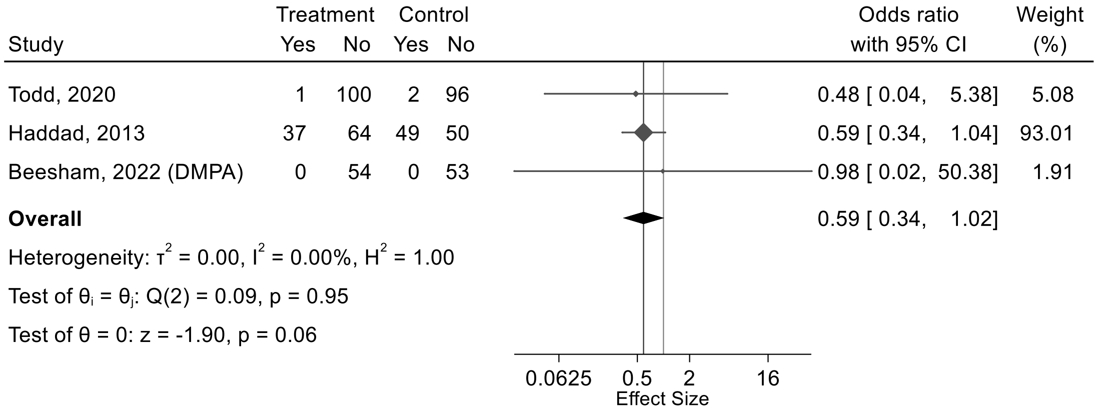


**Supplementary Figure 5.** A) Forest plot presenting the random-effects meta-analysis odds ratios of randomized controlled trials reporting the impact of hormonal contraceptives (treatment) compared to non-hormonal contraceptives (control) on self-reported weight loss including only the LNG-IUD arm for Beesham, 2022. B) Forest plot presenting the random-effects meta-analysis odds ratios of randomized controlled trials reporting the impact of hormonal contraceptives (treatment) compared to non-hormonal contraceptives (control) on self-reported weight loss including only the DMPA arm for Beesham, 2022
